# RNF128 regulates the adaptive metabolic response to fasting by modulating PPARα function

**DOI:** 10.1038/s41418-025-01579-4

**Published:** 2025-09-10

**Authors:** Yu-Lung Lin, Pei-Yao Liu, Yu-Ling Tsai, Chien-Ming Lin, Yu-Guang Chen, Jun-Ren Sun, Yu-Chan Chang, Wen-Chiuan Tsai, Yi-Xuan Ding, Chi-Wei Liu, Shih-Yun Wang, Ying-Chuan Chen

**Affiliations:** 1 The Ph.D. Program for Translational Medicine, College for Medical Science and Technology, Taipei Medical University, Taipei, Taiwan; 2 International Ph.D. Program for Translational Science, College of Medical Science and Technology, Taipei Medical University, Taipei, Taiwan; 3 Graduate Institute of Physiology, College of Biomedical Sciences, National Defense Medical University, Taipei, Taiwan, Republic of China; 4 Graduate Institute of Biodefense, College of Biomedical Sciences, National Defense Medical University, Taipei, Taiwan, Republic of China; 5 Department of Pathology, Tri-Service General Hospital, Taipei, Taiwan, Republic of China; 6 School of Nutrition and Health Sciences, College of Nutrition, Taipei Medical University, Taipei, Taiwan; 7 Department of Pediatrics, Tri-Service General Hospital, National Defense Medical University, Taipei, Taiwan, Republic of China; 8 Division of Hematology/Oncology, Department of Internal Medicine, Tri-Service General Hospital, National Defense Medical University, Taipei, Taiwan, Republic of China; 9 Cancer Institute, University College London, London, UK; 10 Department of Biomedical Imaging and Radiological Sciences, National Yang Ming Chiao Tung University, Taipei, Taiwan; 11 Graduate Institute of Medical Sciences, National Defense Medical University, Taipei, Taiwan, Republic of China; 12 Division of Translational Medicine, Department of Research and Development, Taoyuan General Hospital, Ministry of Health and Welfare, Taoyuan, Taiwan

**Keywords:** Metabolic disorders, Nutrition disorders

## Abstract

Peroxisome proliferator-activated receptor alpha (PPARα) is a crucial transcriptional factor that regulates fatty acid β-oxidation and ketogenesis in response to fasting. However, the mechanisms underlying PPARα function remain unclear. This study identified a novel PPARα-binding protein—RING finger protein 128 (RNF128)—that facilitates PPARα polyubiquitination, resulting in the degradation and suppression of PPARα function during fasting. Furthermore, RNF128 overexpression inhibited fibroblast growth factor 21 expression and lipid metabolism-related genes by facilitating PPARα degradation during fasting. In contrast, silencing RNF128 expression enhanced PPARα-dependent fatty acid β-oxidation and ketogenesis in starved cells. In vivo experiments demonstrated that RNF128 deficiency also significantly reduced lipid levels while increasing fatty acid β-oxidation and ketogenesis during fasting. Adeno-associated virus serotype 8-mediated RNF128 overexpression resulted in increased lipid levels and decreased expression of genes related to fatty acid β-oxidation and ketogenesis in fasted mice. Our findings revealed that RNF128 is crucial for metabolic adaptation to fasting in the liver by interacting with PPARα, thereby enhancing its polyubiquitination and degradation. Therefore, RNF128 is a novel regulator of PPARα function under nutrient-deprived conditions.

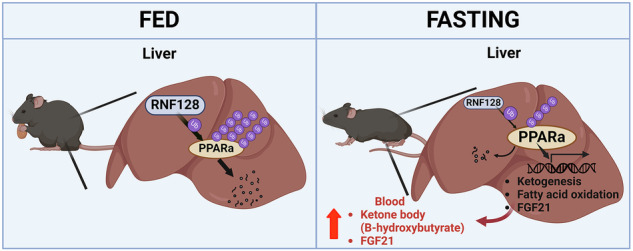

## Introduction

The liver plays a crucial role in regulating whole-body energy metabolism and facilitating communication between metabolic tissues, including adipose tissues and muscles. During fasting, the liver provides energy molecules, including triglycerides and glucose, to extrahepatic tissues for their metabolic activities. Fatty acids derived from adipocytes through lipolysis are used for β-oxidation and ketogenesis in the liver, producing ketone bodies, which serve as primary energy sources for other tissues during fasting [1, 2].

The adaptive metabolic response to fasting is mediated by transcription factors, including peroxisome proliferator-activated receptor alpha (PPARα), cAMP-responsive element binding protein 1, and glucocorticoid receptor [3, 4]. PPARα—a ligand-activated transcription factor—belongs to the nuclear hormone receptor superfamily. It is abundantly expressed in metabolically active tissues, including the liver, skeletal muscle, and brown adipose tissue [5, 6]. PPARα regulates fatty acid β-oxidation and ketogenesis, facilitating an adaptive metabolic response to fasting [7–10]. PPARα-deficient mice exhibit impaired metabolic adaptation, resulting in hyperlipidemia and hypoketonemia during fasting [7, 11]. The PPARα target gene—fibroblast growth factor 21 (FGF21)—primarily expressed in the liver, regulates energy homeostasis by controlling fatty acid β-oxidation and ketogenesis during nutrient deprivation [12–15]. Therefore, the PPARα–FGF21 axis represents a crucial regulator of metabolic adaptation to fasting. During energy deprivation, fine-tuning of PPARα function and activity is crucial for metabolic remodeling. Additionally, post-translational modifications of PPARα, such as ubiquitination, can regulate its function [16]; however, the underlying regulation mechanisms remain unclear.

RING finger protein 128 (RNF128)—a type I transmembrane E3 ligase—regulates T cell tolerance and regulatory T cell function [17, 18]. It is expressed in various organs and mediates interleukin-2 production by inhibiting RhoA GTPase activity and regulating cytoskeletal organization [19]. RNF128 deficiency results in impaired immunotolerance and enhanced susceptibility to autoimmune diseases [20]. Moreover, it is crucial for controlling macrophage activation and neutrophil infiltration during the progression of acute lung injury [21]. RNF128 affects hepatic steatosis and adipocyte differentiation by regulating the levels and activity of sirtuin 1 (SIRT1) and PPARγ, respectively [22, 23]. It also affects apoptosis and cell cycle arrest by interacting with proteins, such as p53 and p21 [24]. Therefore, RNF128 plays a significant role in both immune regulation and metabolic processes.

This study aimed to assess the role of RNF128 in the adaptive metabolic response to fasting using adeno-associated virus serotype 8 (AAV8)-mediated RNF128 overexpression and RNF128 knockout (KO) mice. Our findings indicated that RNF128 is crucial for regulating fasting-induced fatty acid β-oxidation and ketogenesis in the liver by modulating PPARα polyubiquitination and degradation. This highlights a novel role of RNF128 during nutrient deprivation.

## Results

### RNF128 is downregulated in the livers of fasted mice and starved liver cells

RNF128 affects adipocyte differentiation and hepatic steatosis [22, 23]. To assess its role in metabolic flexibility in the liver, RNF128 levels in liver tissues under nutrient-deprived conditions were assessed. We observed reduced RNF128 levels in fasting mice livers (Fig. S1A–C), along with reduced RNF128 mRNA and protein levels in murine primary hepatocytes from fasting mice (Fig. S1D–F). Additionally, both RNF128 mRNA and protein levels were significantly reduced in starved AML12 cells (Fig. S1G–I).

### RNF128 affects serum glucose, serum lipid, and liver function under fasting conditions

**Fig. 1:**
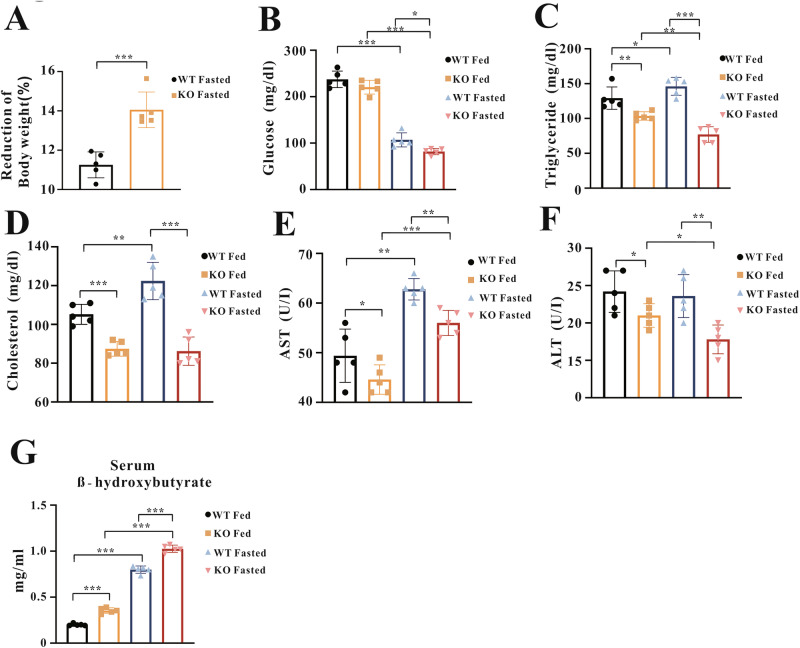
Deletion of RING finger protein 128 lowers serum glucose and triglyceride contents during fasting. **A** Wild-type and RING finger protein 128 knockout mice (8–10 weeks old) are either fed normally or fasted for 24 h, and weight loss is measured (*n* = 5 per group). **B**–**G** Analysis of serum glucose, triglyceride, cholesterol, aspartate aminotransferase, alanine aminotransferase, and serum β-hydroxybutyrate in the indicated groups following either ad libitum feeding or a 24-h fasting period (*n* = 6 per group). Data are presented as the mean ± standard deviation. One-way analysis of variance using the Newman–Keuls post hoc test or Student’s *t-*test is used to assess the statistical significance. ^*^P < 0.05; ^**^P < 0.01; and ^***^P < 0.001.

**Fig. 2:**
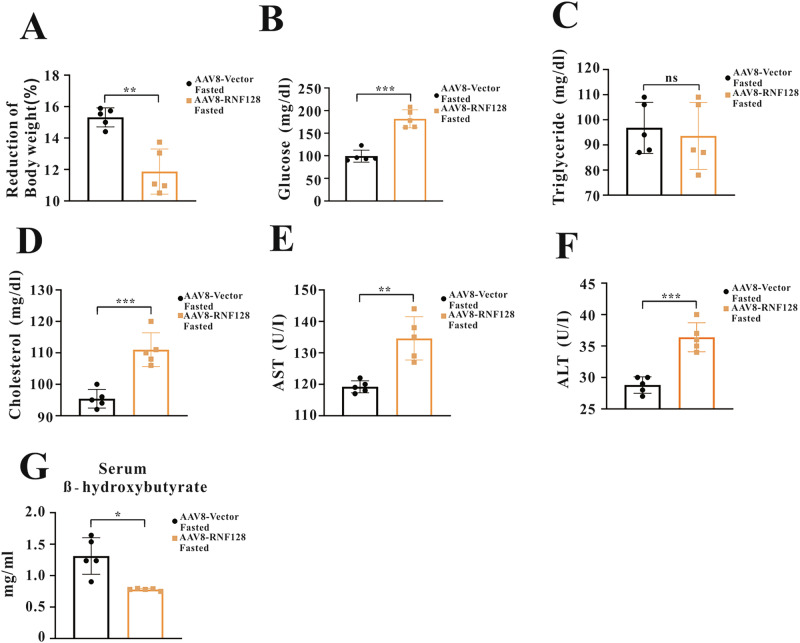
Adeno-associated virus serotype 8-mediate RING finger protein 128 overexpression increases serum glucose and triglyceride contents during fasting. **A** Body weight loss in adeno-associated virus serotype 8-vector and adeno-associated virus serotype 8-RING finger protein 128 mice after 24 h of fasting (*n* = 5 per group). **B**–**G** Alterations in serum glucose, triglyceride, cholesterol, aspartate aminotransferase, alanine aminotransferase, and β-hydroxybutyrate levels obtained from indicated groups after fasting for 24 h (*n* = 5 per group). Data are presented as the mean ± standard deviation. One-way analysis of variance using the Newman–Keuls post hoc test or Student’s *t-*test is used to assess the statistical significance. ^*^P < 0.05; ^**^P < 0.01; and ^***^P < 0.001.

To assess the physiological role of RNF128 in regulating adaptive responses to fasting, the RNF128 KO mouse model was used to assess its effect on metabolic remodeling during fasting. Wild-type (WT) and RNF128 KO mice were fasted for 24 h, and RNF128 expression was confirmed (Fig. S2). RNF128 KO mice exhibited greater weight loss and had lower serum glucose and insulin levels compared to WT mice (Fig. 1A, B, S3). Biochemical analysis revealed significantly increased serum triglyceride and total cholesterol levels in the WT mice after fasting. Conversely, their levels were significantly reduced in the fasted RNF128 KO mice (Fig. 1C, D). Serum alanine aminotransferase (ALT) and aspartate aminotransferase (AST) levels—the common indicators of liver injury [25]—were significantly increased in fasted WT mice. However, their levels were reversed in fasted RNF128 KO mice (Fig. 1E, F). We measured serum β-hydroxybutyrate levels, a ketone body that serves as an energy source during fasting [26], in both WT and RNF128 KO mice under fed and fasted conditions. A significant increase in its levels was observed in fasted WT mice, which was further increased in the fasted RNF128 KO mice (Fig. 1G). Furthermore, in contrast to RNF128 KO mice, fasting-induced body weight loss was significantly reduced in AAV8-RNF128 mice compared with that in controls (Fig. 2A). Additionally, AAV8-RNF128 mice exhibited increased serum glucose, ALT, AST, cholesterol, and insulin levels during fasting (Fig. 2B–F, S3). Fasted AAV8-RNF128 mice exhibited lower serum β-hydroxybutyrate levels compared with those of fasted AAV8-vector mice (Fig. 2G). These findings support the regulatory role of RNF128 in the adaptive responses during fasting.

### RNF128 mediates FGF21 induction in vivo

**Fig. 3:**
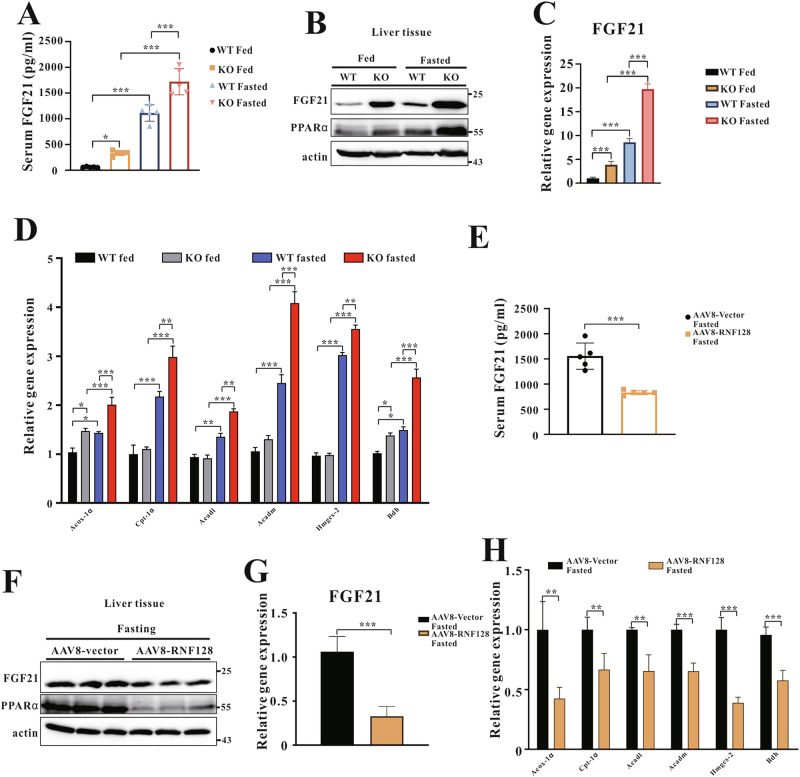
RING finger protein 128 regulates hepatic ketogenesis and fatty acid β-oxidation during nutrient deprivation. **A** Serum fibroblast growth factor 21 (FGF21) concentrations are measured in the indicated groups after ad libitum feeding or 24-h fasting (*n* = 6 per group). **B** Immunoblots of peroxisome proliferator-activated receptor alpha and FGF21 in the liver of mice subjected to ad libitum feeding or 24-h fasting. **C**, **D** The mRNA expressions of FGF21, peroxisomal acyl-coenzyme A oxidase 1, carnitine palmitoyltransferase 1, acyl-coA dehydrogenase long chain, acyl-coA dehydrogenase medium chain, 3-hydroxy-3-methylglutaryl-coA synthase 2, and 3-hydroxybutyrate dehydrogenase in indicated samples were analyzed using quantitative reverse transcription PCR. **E** Serum FGF21 concentrations of adeno-associated virus serotype 8-vector and adeno-associated virus serotype 8-RING finger protein 128 mice after 24-h fasting (*n* = 6 per group). **F** Immunoblots of peroxisome proliferator-activated receptor alpha and FGF21 in indicated samples. **G**, **H** The mRNA expressions of FGF21, peroxisomal acyl-coenzyme A oxidase 1, carnitine palmitoyltransferase 1, acyl-coA dehydrogenase long chain, acyl-coA dehydrogenase medium chain, 3-hydroxy-3-methylglutaryl-coA synthase 2, and 3-hydroxybutyrate dehydrogenase in indicated samples are analyzed using quantitative reverse transcription polymerase chain reaction. Data are presented as the mean ± standard deviation. One-way analysis of variance using the Newman–Keuls post hoc test or Student’s *t-*test is used to assess the statistical significance. ^**^P < 0.01 and ^***^P < 0.001.

FGF21 regulates metabolic flexibility in the liver during fasting [12–15]. We measured FGF21 levels in WT and RNF128 KO mice to assess RNF128 role in this process. Serum FGF21 levels were significantly higher in fasted KO mice than those in fasted WT mice (Fig. 3A), whereas a reduction was observed in fasted AAV8-RNF128 mice (Fig. 3E). Similarly, both FGF21 mRNA and protein levels were significantly higher in the livers of fasted KO mice than those in the controls (Fig. 3B, C). Conversely, FGF21 protein and mRNA levels decreased in the fasted AAV8-RNF128 mice (Fig. 3F, G). Additionally, the mRNA levels of genes associated with fatty acid β-oxidation (peroxisomal acyl-coenzyme A oxidase 1, carnitine palmitoyltransferase 1, acyl-coA dehydrogenase long chain, and acyl-coA dehydrogenase medium chain) and ketogenesis (3-hydroxy-3-methylglutaryl-coA synthase 2, 3-hydroxybutyrate dehydrogenase) were increased in fasted KO mice (Fig. 3D). Furthermore, the mRNA levels of these genes were increased in primary hepatocytes of fasted KO mice (Fig. S4) and significantly decreased in fasted AAV8-RNF128 mice compared with those in the controls (Fig. 3H). These results indicated that RNF128 regulates FGF21 induction and metabolic responses during nutrient deprivation.

### Loss of RNF128 expression increases adaptive metabolic response in starved liver cells

**Fig. 4:**
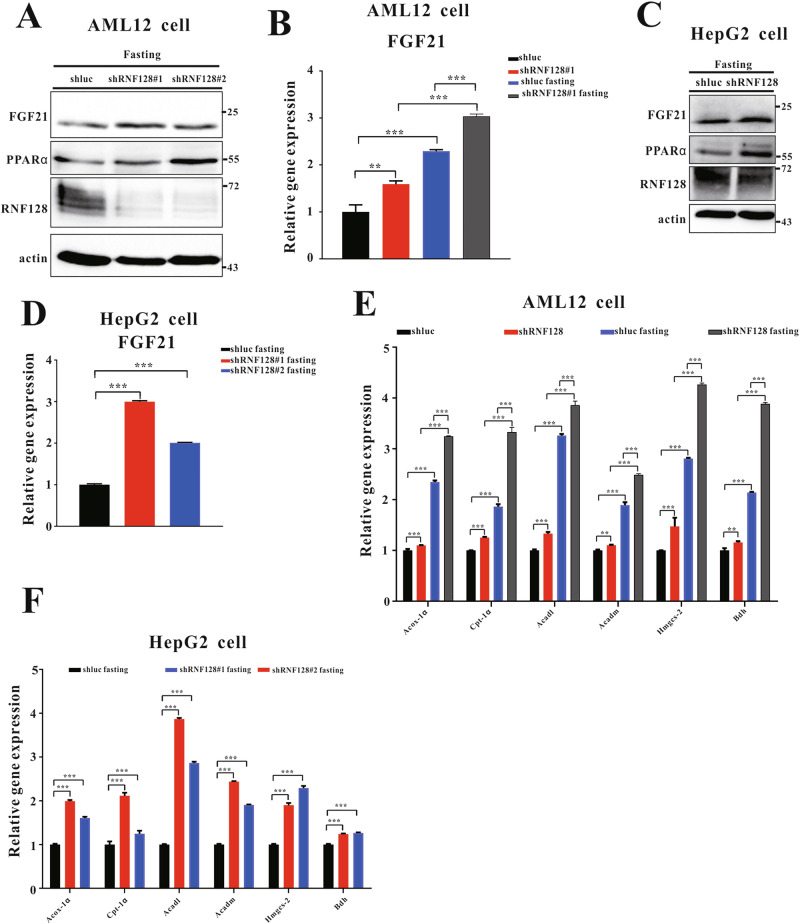
Deletion of RING finger protein 128 (RNF128) enhances peroxisome proliferator-activated receptor alpha functions and facilitates ketogenesis and fatty acid β-oxidation during starvation. **A** Immunoblots of RNF128, proliferator-activated receptor alpha, and fibroblast growth factor 21 (FGF21) in AML12/shluc and AML12/shRNF128 cell lines after treatment with or without fasted medium (containing 1 g/l glucose and 0.5% fetal bovine serum). **B** The mRNA expressions of FGF21 in the indicated samples. **C** Immunoblots of RNF128, proliferator-activated receptor alpha, and FGF21 in HepG2/shluc and HepG2/shRNF128 cell lines subjected to fasted medium. **D** The mRNA expressions of FGF21 in fasted HepG2/shluc and HepG2/shRNF128 cell lines. The mRNA expressions of peroxisomal acyl-coenzyme A oxidase 1, carnitine palmitoyltransferase 1, acyl-coA dehydrogenase long chain, acyl-coA dehydrogenase medium chain, 3-hydroxy-3-methylglutaryl-coA synthase 2, and 3-hydroxybutyrate dehydrogenase (**E**) in AML12/shluc and AML12/shRNF128 or **F** in HepG2/shluc and HepG2/shRNF128 cell lines subjected to a fasted medium for 24 h. Data are presented as the mean ± standard deviation. One-way analysis of variance using the Newman–Keuls post hoc test or Student’s *t-*test is used to assess the statistical significance. ^**^P < 0.01 and ^***^P < 0.001.

To elucidate the relationship between RNF128 and adaptive metabolic responses in vitro, we silenced RNF128 expression in AML12 and HepG2 (AML12/shRNF128 and HepG2/shRNF128) cells and confirmed its downregulation (Fig. 4A, C). FGF21 mRNA and protein levels were higher in starved AML12/shRNF128 cells than those in the controls (Fig. 4A, B). Additionally, the analysis of mRNA levels of fatty acid β-oxidation- and ketogenesis-associated genes indicated that lipid and ketone metabolism-related gene expressions were upregulated in starved AML12/shRNF128 cells compared with those in the controls (Fig. 4E). Similar results were observed in the HepG2/shRNF128 cells (Fig. 4C, D, F). These results indicated that RNF128 deficiency may increase FGF21 induction, thereby enhancing hepatic fatty acid β-oxidation and ketogenesis during fasting.

### RNF128 overexpression attenuates adaptive metabolic response in starved liver cells

**Fig. 5:**
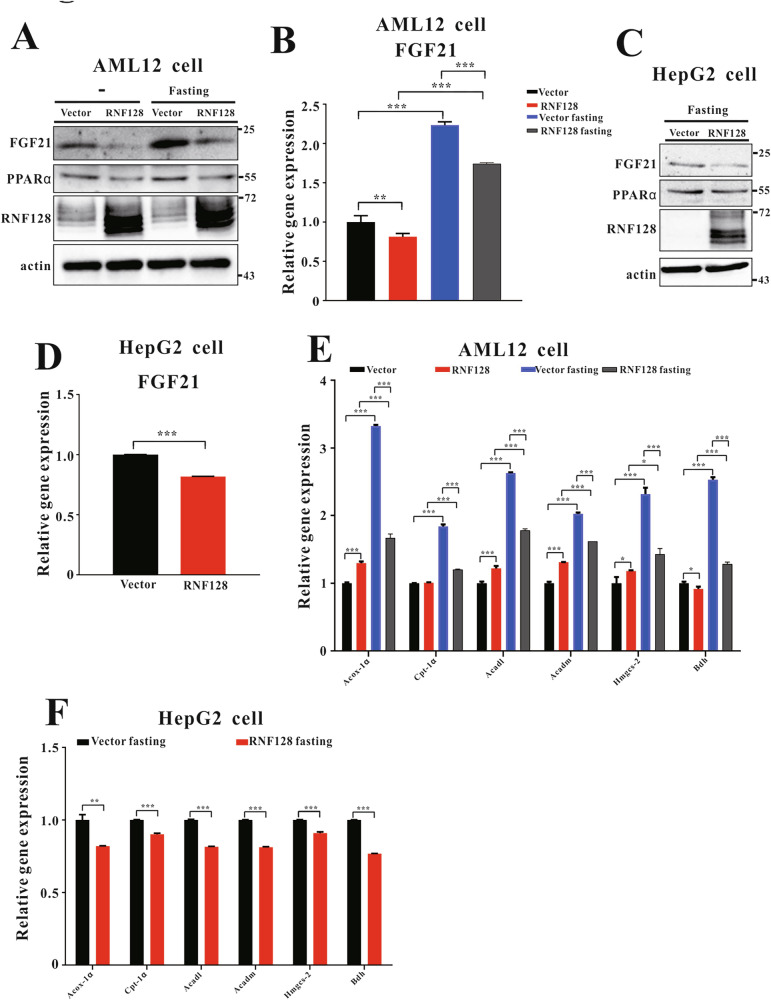
Overexpression of RING Finger Protein 128 (RNF128) reduces peroxisome proliferator-activated receptor alpha functions and attenuates ketogenesis and fatty acid β-oxidation during starvation. **A** Immunoblots of RING finger protein 128 (RNF128), proliferator-activated receptor alpha, and fibroblast growth factor 21 (FGF21) in AML12/vector and AML12/RNF128 cell lines after treatment with or without fasted medium. **B** The mRNA expressions of FGF21 in indicated samples. **C** Immunoblots of RNF128, proliferator-activated receptor alpha, and FGF21 in HepG2/vector and HepG2/RNF128 cell lines with fasted medium. **D** The mRNA expressions of FGF21 in fasted HepG2/vector and HepG2/RNF128 cell lines. The mRNA expressions of peroxisomal acyl-coenzyme A oxidase 1, carnitine palmitoyltransferase 1, acyl-coA dehydrogenase long chain, acyl-coA dehydrogenase medium chain, 3-hydroxy-3-methylglutaryl-coA synthase 2, and 3-hydroxybutyrate dehydrogenase (**E**) in AML12/vector and AML12/RNF128 or **F** in HepG2/vector and HepG2/RNF128 cell lines subjected to fasted medium for 24 h. Data are presented as the mean ± standard deviation. One-way analysis of variance using the Newman–Keuls post hoc test or Student’s *t-*test is used to assess the statistical significance. ^**^P < 0.01 and ^***^P < 0.001.

To further assess the role of RNF128 in the adaptive response to fasting, we established a stable overexpression of RNF128 in AML12 and HepG2 cells and subsequently quantified RNF128 levels (Fig. 5A, C). FGF21 mRNA and protein levels were reduced in starved AML12/RNF128 cells than those in the controls (Fig. 5A, B). Additionally, the mRNA levels of fatty acid β-oxidation- and ketogenesis-associated genes were significantly reduced in starved AML12/RNF128 cells than those in the controls (Fig. 5E). Similar results were observed in HepG2/shRNF128 cells (Fig. 5C, D, F). Therefore, RNF128 could attenuate the metabolic adaptation to fasting in liver cells.

### RNF128 reduces PPARα protein levels

**Fig. 6:**
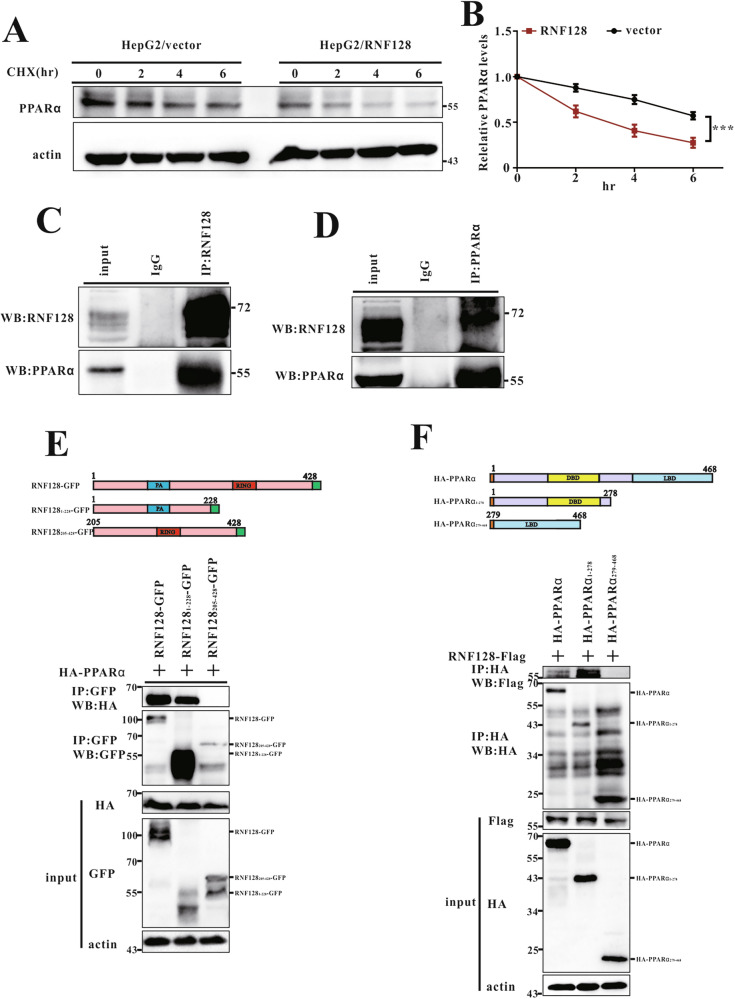
RING finger protein 128 interacts with proliferator-activated receptor alpha. **A**, **B** HepG2/vector and HepG2/RNF128 cells are treated with cycloheximide (100 g/mL) and harvested at the indicated times. Cell lysates are subjected to immunoblotting with the indicated antibodies. **C**, **D** Endogenous RNF128 interacts with endogenous PPARα. Extracts from AML-12 cells are prepared, immunoprecipitated with anti-RNF128, anti-PPARα, or rabbit anti-immunoglobulin G antibodies, and analyzed using anti-RNF128 and anti-PPARα antibodies. **E** HEK293 cells are transiently transfected with HA-PPARα, RNF128-GFP, RNF128_1–228_-GFP, and RNF128_205–428_-GFP after 48 h. Lysates are harvested and subjected to immunoprecipitation with GFP antibody and analyzed by immunoblotting with the indicated antibody. **F** HEK293 cells are transiently transfected with Flag-RNF128, HA-PPARα, HA-PPARα_1–278_, and HA-PPARα_279–468_ after 48 h. Lysates are harvested and subjected to immunoprecipitation with HA antibody and analyzed by immunoblotting with the indicated antibody. Data are presented as the mean ± standard deviation. One-way analysis of variance using the Newman–Keuls post hoc test or Student’s *t-*test is used to assess the statistical significance. ^***^P < 0.001.

PPARα plays a crucial role in regulating hepatic FGF21 expression, which regulates fatty acid β-oxidation and ketogenesis in the liver during food deprivation [27]. NGS and QPCR analysis showed increased PPARα levels during fasting (Fig. S5), and RNF128 was found to regulate both FGF21 mRNA and protein levels in fasted liver cells (Figs. 3–5). Thus, we measured PPARα expression in the liver. We observed a significant increase in PPARα levels in the livers of RNF128 KO mice compared with those in the WT mice, regardless of the fed or fasted state (Fig. 3B). Conversely, PPARα levels were notably reduced in the livers of fasted AAV8-RNF128 mice compared with those of fasted AAV8-vector mice (Fig. 3F). To further assess the regulatory role of RNF128 on PPARα levels, we measured the PPARα levels in AML12 and HepG2 cells overexpressing RNF128. RNF128 overexpression significantly reduced the endogenous PPARα levels (Fig. 5A, C). Conversely, stable RNF128 knockdown in AML12 and HepG2 cells increased PPARα levels (Fig. 4A, C). To determine whether the reduction in PPARα levels was because of reduced protein stability, we used cycloheximide, an inhibitor of de novo protein synthesis. Our results revealed that RNF128 regulates PPARα protein levels by reducing its stability (Fig. 6A, B). Therefore, RNF128 modulates PPARα levels, potentially contributing to cellular metabolic flexibility during fasting.

### RNF128 interacts with PPARα and facilitates its ubiquitination

**Fig. 7:**
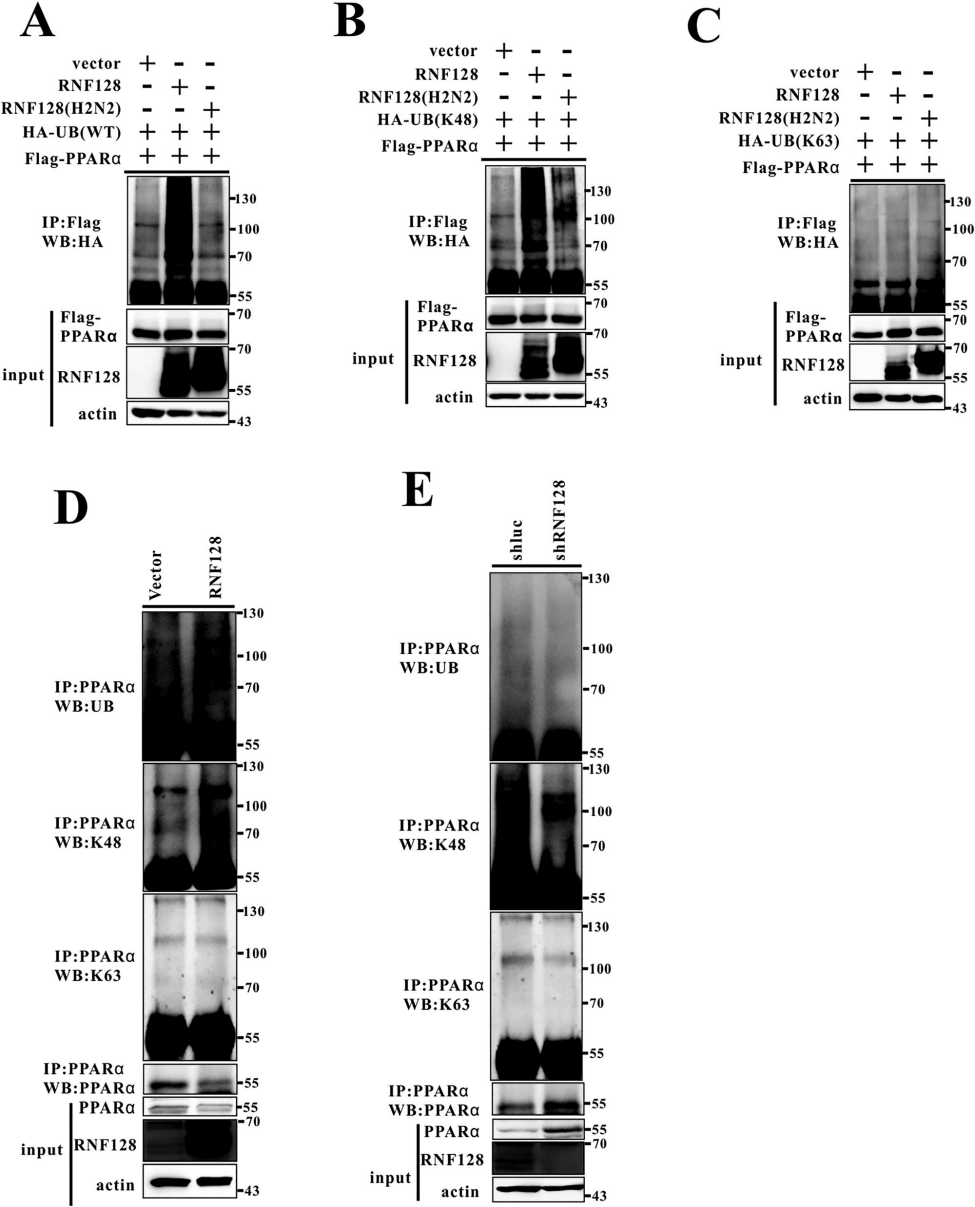
RING finger protein 128 promotes polyubiquitination of proliferator-activated receptor alpha. **A**–**C** HEK293 cells are transiently transfected with hemagglutinin-ubiquitin (wild-type), hemagglutinin-ubiquitin (K48), hemagglutinin-ubiquitin (K63), Flag-PPARα, and RNF128 after 48 h. Lysates are harvested and subjected to immunoprecipitation with Flag antibody and analyzed for ubiquitylation by immunoblotting with the indicated antibody. **D**, **E** Lysates from HepG2 stable cell lines with RNF128 overexpression or silencing are immunoprecipitated with PPARα antibody and analyzed for ubiquitylation by immunoblotting with the specified antibody.

We assessed the mechanisms through which RNF128 regulates PPARα expression. Through co-immunoprecipitation and mass spectrometry analysis of proteins bound to PPARα, we found RNF128 to be the most abundant E3 ligase protein interacting with PPARα (Fig. S6). Using AML12 cells, we confirmed the interaction between RNF128 and PPARα through a co-immunoprecipitation assay. A reciprocal interaction between RNF128 and PPARα was observed in the AML12 cells (Fig. 6C, D). To evaluate the interaction between RNF128 and PPARα during fasting, a significant decrease was observed (Fig. S7). Additionally, we examined the subcellular localization of endogenous RNF128 and PPARα in the AML12 cells. RNF128 co-localized with PPARα in the nucleus (Fig. S8A), and confocal imaging of the HepG2 cells demonstrated similar results (Fig. S8B). RNF128 consists of two highly conserved domains, namely the N-terminal protease-associated (PA) domain and the RING finger domain. To identify the specific region of RNF128 that interacts with PPARα, we generated truncated variants of RNF128 (RNF128_1–228_ and RNF128_205–428_). Our findings indicated that the truncated form RNF128_1–228_, which includes the PA domain, is responsible for binding to PPARα (Fig. 6E). We further generated a series of PPARα truncations: PPARα_1–278_ and PPARα_279–468_. Co-IP experiments demonstrated that the truncated form PPARα_1–278_, which includes the DNA binding domain (DBD), was crucial for the interaction with RNF128 (Fig. 6F). As RNF128 is an E3 ubiquitin ligase that enhances the ubiquitylation of target proteins [21, 23, 24, 28], we hypothesized that RNF128 directly facilitates PPARα ubiquitination. Consequently, we transfected HEK293 cells with plasmids encoding RNF128, ubiquitin, and PPARα and analyzed their expression. RNF128 and PPARα co-expression significantly enhanced PPARα polyubiquitination (Fig. 7A). Additionally, we investigated the ubiquitination of endogenous PPARα within the cells. RNF128 overexpression enhanced PPARα polyubiquitination compared to that in the controls (Fig. 7D). In contrast, RNF128 knockdown significantly reduced PPARα polyubiquitination (Fig. 7E). To further assess RNF128-mediated polyubiquitination of PPARα, we compared WT ubiquitin with mutant ubiquitin containing a single lysine residue (K48 or K63). RNF128 significantly enhanced PPARα polyubiquitination in the presence of hemagglutinin-tagged ubiquitin K48 (HA-Ub-K48), but not with K63 (Fig. 7A–C). Additionally, enhanced K48-associated PPARα ubiquitination was observed in RNF128 overexpressing AML12 cells (Fig. 7C), whereas RNF128 silencing reversed the K48-associated PPARα ubiquitination (Fig. 7D). These findings indicated that RNF128 mediates K48-associated PPARα polyubiquitination.

### PPARα is required for RNF128-mediated adaptive metabolic response to fasting

**Fig. 8:**
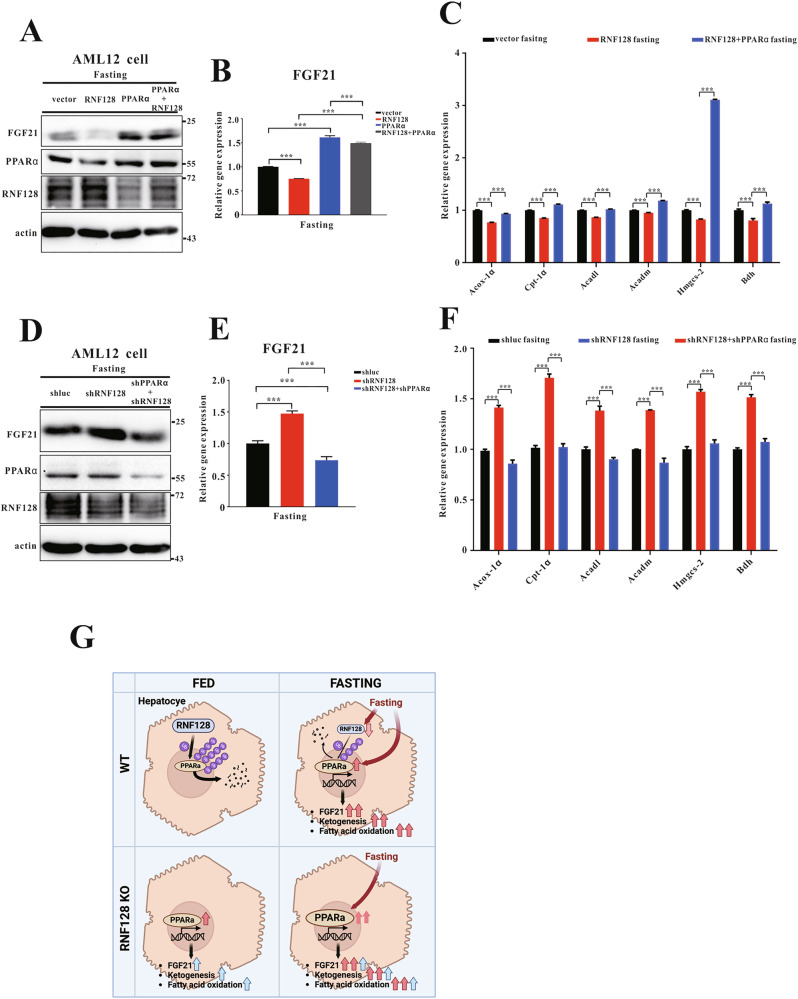
RING finger protein 128 mediates proliferator-activated receptor alpha-dependent ketogenesis and fatty acid β-oxidation during fasting. **A** Immunoblot analysis of RING finger protein 128, proliferator-activated receptor alpha, and fibroblast growth factor 21 (FGF21) expression in indicated stable cell lines treated with fasted medium. **B**, **C** The mRNA expressions of FGF21, peroxisomal acyl-coenzyme A oxidase 1, carnitine palmitoyltransferase 1, acyl-coA dehydrogenase long chain, acyl-coA dehydrogenase medium chain, 3-hydroxy-3-methylglutaryl-coA synthase 2, and 3-hydroxybutyrate dehydrogenase in indicated samples. **D** Immunoblots of RING finger protein 128, proliferator-activated receptor alpha, and FGF21 in indicated stable cell lines subjected to fasted medium. **E**, **F** The mRNA expressions of FGF21, peroxisomal acyl-coenzyme A oxidase 1, carnitine palmitoyltransferase 1, acyl-coA dehydrogenase long chain, acyl-coA dehydrogenase medium chain, 3-hydroxy-3-methylglutaryl-coA synthase 2, and 3-hydroxybutyrate dehydrogenase in indicated samples. **G** The working model of RNF128 in the regulation of the adaptive metabolic response to fasting. The figure is created using elements from BioRender.com. Data are presented as the mean ± standard deviation. One-way analysis of variance using the Newman–Keuls post hoc test or Student’s *t-*test is used to assess the statistical significance. ^***^P < 0.001.

To assess the effect of RNF128 on PPARα-dependent metabolic response during fasting, we overexpressed RNF128, PPARα, or both in HepG2 cells and analyzed FGF21 levels after nutrient starvation. RNF128 overexpression reduced FGF21 mRNA and protein levels in starved HepG2 cells, whereas their increase was observed in the HepG2/RNF128/PPARα cell lines (Fig. 8A, B). Consistently, the mRNA levels of fatty acid β-oxidation- and ketogenesis-associated genes exhibited similar patterns (Fig. 8C). Interestingly, the increased FGF21 levels were restored in HepG2/shRNF128 cells upon PPARα silencing (Fig. 8D, E). Additionally, the increased mRNA levels of fatty acid β-oxidation- and ketogenesis-associated genes were eliminated in HepG2/shRNF128/shPPARα cells (Fig. 8F). These findings indicated that the RNF128-mediated regulation of PPARα plays a significant role during fasting.

## Discussion

During fasting, hepatic PPARα—a primary regulator of energy homeostasis—modulates target genes associated with lipid catabolism and ketogenesis to regulate metabolism flexibility [9]. Identifying the novel regulators that mediate PPARα function during fasting is imperative. This study, for the first time, reports that RNF128 plays a crucial role in regulating metabolic adaptation to fasting. RNF128 expression was significantly increased in the livers of fasted mice and starved liver cells. RNF128 modulated PPARα function both in vitro and in vivo. RNF128 deficiency enhanced fasting-induced fatty acid β-oxidation and ketogenesis, thereby improving lipid and ketone metabolism during nutrient deprivation. In contrast, AAV8-mediated hepatic overexpression of RNF128 resulted in the opposite phenotype. Mechanistically, RNF128 negatively regulated PPARα expression by facilitating its polyubiquitination and degradation (Fig. 8G).

PPARα is a primary transcription factor involved in regulating the expression of genes associated with lipid metabolism and ketogenesis during fasting. Our findings revealed that fasting induced PPARα expression, whereas RNF128 expression was downregulated in the liver cells (Fig. S1G). Additionally, PPARα overexpression inhibited RNF128 expression in AML12 cells (Fig. S9A). In contrast, RNF128 expression was upregulated in PPARα-silenced cells (Fig. S9B). To further examine whether RNF128 is a PPARα target gene, three potential PPARα-responsive elements were identified in the promoter region of the RNF128 gene (Fig. S10). DNA-affinity precipitation assays (DAPAs) were conducted using these PPARα-responsive elements of RNF128 (Fig. S10A), indicating that PPARα can bind to all three elements in the RNF128 promoter region. This observation was supported by chromatin immunoprecipitation (ChIP) analysis, which showed that PPARα interacted with responsive elements 1, 2, and 3 of the RNF128 promoter (Fig. S10B). Therefore, RNF128 may be a target of PPARα, potentially forming a feedback loop that regulates metabolic adaptation to fasting.

Our study revealed that RNF128 regulates liver metabolism by inhibiting the PPARα signaling pathway that reduces fatty acid β-oxidation and ketogenesis. Various PPARα target genes, specifically FGF21, play crucial physiological roles in lipid catabolism, ketogenesis, and obesity [15, 29]. Additionally, fasting-induced hepatic PPARα expression was mediated by the inhibition of E3 ligase RNF128 expression, thereby enhancing FGF21 induction. In contrast, enhanced RNF128 expression resulted in PPARα polyubiquitination and degradation, subsequently reducing FGF21 levels. Therefore, the PPARα–FGF21 axis is a significant target of RNF128 that regulates metabolism during nutrient deprivation.

FGF21 mRNA and protein levels were increased in the livers of RNF12-deficient mice. Similarly, serum FGF21 levels increased in RNF128 KO mice during fasting (Fig. 3A–C). In contrast, RNF128 overexpression in mice and liver cells exerted the opposite effect (Fig. 4A–C). FGF21, a hepatokine, plays a significant role in the regulation of glucose and lipid homeostasis. As FGF21 induction can be controlled by RNF128, its effects on metabolism may be mediated through FGF21 regulation. Notably, further studies are required to explore the direct functional interaction between RNF128 and FGF21 in the regulation of glucose and lipid metabolism.

Recently, FGF21 was identified to be crucial in treating hepatic steatosis and may serve as a potential therapeutic target for nonalcoholic fatty liver disease [30, 31]. RNF128 can contribute to the development of hepatic steatosis by inhibiting SIRT1 [22]. Additionally, SIRT1 can induce hepatic FGF21 expression to prevent liver steatosis during fasting [32]. However, whether the interaction between RNF128 and SIRT1 affects the role of FGF21 in regulating adaptive metabolic responses remains unclear. Further studies are required to explore this relationship.

Ubiquitination and deubiquitination are reversible processes that modify the behavior of target proteins, influencing their stability, intracellular localization, and enzyme activity [33]. Recent studies have shown that USP25 can interact with and deubiquitinate PPARα, thereby stabilizing its stability and improving hepatic steatosis and metabolic dysfunction associated steatotic liver disease (MASLD) induced by a high-fat diet in mice [34, 35]. To investigate the involvement of USP25 in the RNF128-PPARα regulatory axis, the ubiquitination status of PPARα was examined in the presence of USP25. HEK293 cells were transfected with plasmids encoding RNF128, USP25, ubiquitin, and PPARα, and their expression was analyzed. RNF128 increased PPARα polyubiquitination, while co-expression of USP25 with RNF128 decreased PPARα polyubiquitination (Fig. S11). Further research into the interactions between USP25 and RNF128 in PPARα regulation may provide additional information.

In summary, our study identified RNF128 as a potential regulator of the PPARα–FGF21 axis involved in metabolic adaptation during fasting. We elucidated the underlying mechanisms and provided further insights. We believe that our findings offer a novel strategy for the management of metabolic disorders.

## Materials and Methods

### Animal experiments

The RNF128 KO mice, generated on a C57B/6J background via CRISPR–Cas9, exhibit a deletion at the exon 1 start codon. These mice were generated using the Transgenic Mouse Model Core (Taipei, Taiwan) [23]. The mice were housed under a standard 12:12-h light/dark cycle at 22 ± 1 °C. For the fasting experiment, 8-week-old male WT and RNF128 KO mice were used. The mice had ad libitum access to food for 24 h, whereas the fasted mice were deprived of food for 24 h and euthanized simultaneously with the fed mice. For in vivo AAV administration, AAV8-vector and AAV8-RNF128 were injected into the tail vein at a dose of 1 × 10^12^ vg/mouse in a total volume of 200 µl into 7-week-old male C57BL/6J mice.

### Cells, plasmids, and transfection procedures

HepG2 cells were cultured in Dulbecco’s modified Eagle’s medium supplemented with 10% fetal bovine serum (FBS). AML12 cells were grown in a 1:1 mixture of Dulbecco’s modified Eagle’s medium high glucose and Ham’s F-12 supplemented with 10% FBS. To mimic fasting conditions, cells at a confluence of 70–80% were cultured in a fasting medium supplemented with 1 g/L glucose and 0.5% FBS [36]. RNF128 was cloned into a pCMV-TNT vector (Promega, CA, USA) using EcoRI and BamHI restriction sites. FLAG-PPARα, HA-ubiquitin-WT, HA-ubiquitin-48, and HA-ubiquitin-K63 plasmids were purchased from Addgene (Watertown, MA, USA). Transfection was performed using jetPRIME (New York, USA), following the manufacturer’s protocol. Cells were plated at a low density (approximately 1 × 10^5^ cells/60 mm dish) and allowed to grow to 50–60% confluence. Subsequently, jetPRIME-mediated gene transfection was performed, and the transfected cells were lysed using the radioimmunoprecipitation assay buffer.

### Primary hepatocyte isolation and culture

Primary hepatocytes were isolated from WT or RNF128 KO mice that were either fed or fasted. The liver was perfused through the inferior vena cava with a perfusate outflow. Initially, the liver was perfused with 100 ml of phosphate-buffered saline containing 0.5 mM ethylenediaminetetraacetic acid, followed by 25 ml of phosphate-buffered saline with 0.5 mM ethylenediaminetetraacetic acid and 0.1% collagenase type IV (Sigma-Aldrich, St. Louis, MO, USA). The liver lobes were transferred to a culture dish containing William’s E Medium (GIBCO, Thermo Fisher Scientific, Waltham, MA, USA) and torn into pieces using forceps. The hepatic cells were filtered through 70-um filters into 50-ml tubes and centrifuged (50 × *g*, 3 min, 4 °C). The resulting hepatocyte pellet was washed twice with William’s E medium. Finally, the hepatocytes were resuspended in William’s E medium containing 10% FBS. Following incubation at 37 °C with 5% CO_2_ for 3 h, the hepatocytes were washed to remove dead cells, and a fresh culture medium was added.

### Immunoprecipitation and immunoblot analysis

Cells were lysed in lysis buffer containing 50 mM Tris (pH 8.0), 5 mM NaCl, 0.5% NP-40, and 1× protease inhibitor, subjected to three freeze/thaw cycles, and proteins were extracted. Protein concentration was determined using the Bradford method (Bio-Rad, CA, USA). The cell extracts containing equivalent amounts of proteins were immunoprecipitated in lysis buffer containing polyclonal antibodies against RNF128, PPARα, GFP, or FLAG at 4 °C overnight. Dynabeads™ Protein G (Invitrogen, Waltham, MA, USA) were added to the immunoprecipitation mixture for 1 h before three washes with SNNTE buffer (5% sucrose, 1% NP-40, 0.5 M NaCl, 50 mM Tris [pH 7.4], and 5 mM ethylenediaminetetraacetic acid). Subsequently, the precipitate was suspended in sodium dodecyl sulfate (SDS)–polyacrylamide gel electrophoresis sample buffer, boiled, and loaded onto an SDS–polyacrylamide gel. The separated proteins were transferred onto a nitrocellulose membrane and blocked for 1 h in 10 mM Tris (pH 7.6), 137 mM NaCl, and 0.1% (v/v) Tween 20 containing 5% skimmed milk. The membranes were incubated overnight at 4 °C with the specified primary antibodies, followed by incubation with a secondary antibody (horseradish peroxidase-conjugated anti-mouse or anti-rabbit IgG). Detection was performed using enhanced chemiluminescence reagents (GE Healthcare, Chicago, IL, USA). The following primary antibodies were used: anti-PPARα (15540-1-AP; Proteintech, Rosemount, IL, USA), anti-HA (51064-2-AP; Proteintech), anti-GFP (SC-9996, Santa Cruz, USA), anti-FLAG (F1804; MilliporeSigma, Burlington, MA, USA), anti-FGF21 (JA10-97; Invitrogen), anti-ubiquitin (P4D1; Cell Signaling, Danvers, MA, USA), anti-K48 ubiquitin (D9D5; Cell Signaling), anti-K63 ubiquitin (D7A11; Cell Signaling), anti-actin (MAb1501; Chemicon, Rolling Meadows, IL, USA), and anti-RNF128 (prepared in our laboratory).

### In vivo ubiquitination assays

HepG2/RNF128 or HepG2/shRNF128 cells were lysed in the lysis buffer (50 mM Tris [pH 8.0], 5 mM NaCl, 0.5% NP-40, and 1× protease inhibitor) and subjected to three freeze/thaw cycles for protein recovery. Equivalent amounts of protein were immunoprecipitated overnight at 4 °C using an anti-PPARα antibody in the lysis buffer. Dynabeads Protein G (Invitrogen) were added to the immunoprecipitation mixture and incubated at 4 °C for 1 h. Samples were washed thrice with SNNTE buffer, resuspended in SDS–polyacrylamide gel electrophoresis sample buffer, and loaded onto an SDS–polyacrylamide gel. Subsequently, the gel was transferred onto a nitrocellulose membrane and probed with the specified primary antibodies. The proteins of interest were detected using enhanced chemiluminescence reagents (GE Healthcare).

### Virus particle production, viral transduction, and RNA interference

RNF128 was cloned into a pQCXIP vector (Clontech Laboratories, Mountain View, CA, USA). The pQCXIP-RNF128 and pQCXIP-empty plasmids were transfected into GP2-293 cells using jetPRIME® (Polyplus, NY, USA). Short hairpin RNA (shRNA) oligonucleotides were cloned into the expression vector, pSIREN-Retro-Q (Clontech Laboratories) (RNF128 shRNA target sequence 1: 5′-GAGGCATCCAAGTCACAATGG-3′; RNF128 shRNA target sequence 2: 5′-GCAGGAAGCAGAGGCAGTTAA-3ʹ). The cells were infected with the specified retroviruses in a selection medium containing 2 μg/ml polybrene. After 48 h of infection, puromycin-resistant clones were selected by treating the cells with 2 μg/ml puromycin. The AAV expression vector was constructed using the Helper Free Expression System (Cell Biolabs Inc., San Diego, CA, USA). RNF128 or PPARα was cloned into the pAAV-MCS vector. AAV8 overexpressing RNF128 or PPARα was generated following the standard protocols. The shRNA oligonucleotides were cloned into an AAV shRNA expression vector (pAAV-U6-GFP). AAVs overexpressing PPARα shRNA were generated following the standard protocols (PPARα shRNA target sequence 1: 5′- CCCTTATCTGAAGAATTCT-3′; PPARα shRNA target sequence 2: 5′-CCCTTATCTGAAGAATTCT-3′).

### Biochemical analysis

Serum triglyceride, cholesterol, ALT, and AST levels were quantified using commercially available kits from FUJIFILM (TG-P III, TCHO-P III, GPT/ALT-P III, and GOT/AST-P III, respectively). Serum FGF21 levels were assessed using an ELISA kit from R&D Systems (Catalog#: MF2100). Serum β-OH butyrate was analyzed colorimetrically using a Cayman Chemical kit (Catalog#: 700190).

### Quantitative reverse-transcription PCR

RNA was isolated from cells and tissues using the TRIzol reagent (Sigma, St. Louis, MO, USA). Complementary DNAs were synthesized using Epicenter MMLV. Gene expression in cells was analyzed using the Applied Biosystems 7500 Real-Time PCR System and IQ2 FAST qPCR kit. The gene expressions in tissues were analyzed using a Roche LightCycler 480. The primers used are listed in Supplementary Table 1.

### Statistical analysis

Data analysis and graphing were performed using GraphPad Prism 7 (GraphPad Software). All data were expressed as the mean ± standard deviation. One-way analysis of variance with multiple comparative analyses was used to compare multiple datasets. An unpaired two-tailed Student’s *t*-test was used to analyze two datasets. Statistical significance was set at *P* < 0.05.

## Supplementary information

SUPPLEMENTAL MATERIAL

uncropped original western blots

## Data Availability

The experimental data sets generated and/or analyzed during the current study are available from the corresponding author upon reasonable request. No applicable resources were generated during the current study.
