# Supplementary material for: RNF128 regulates the adaptive metabolic response to fasting by modulating PPARα function
Source: Cell Death Differ. 2025 Sep 10;33(3):512–24. doi: 10.1038/s41418-025-01579-4 (PMC13035816; doi:10.1038/s41418-025-01579-4)
Supplement: Supplementary file 1 — SUPPLEMENTAL MATERIAL [file 41418_2025_1579_MOESM1_ESM.docx]

**Supplementary Information**

**
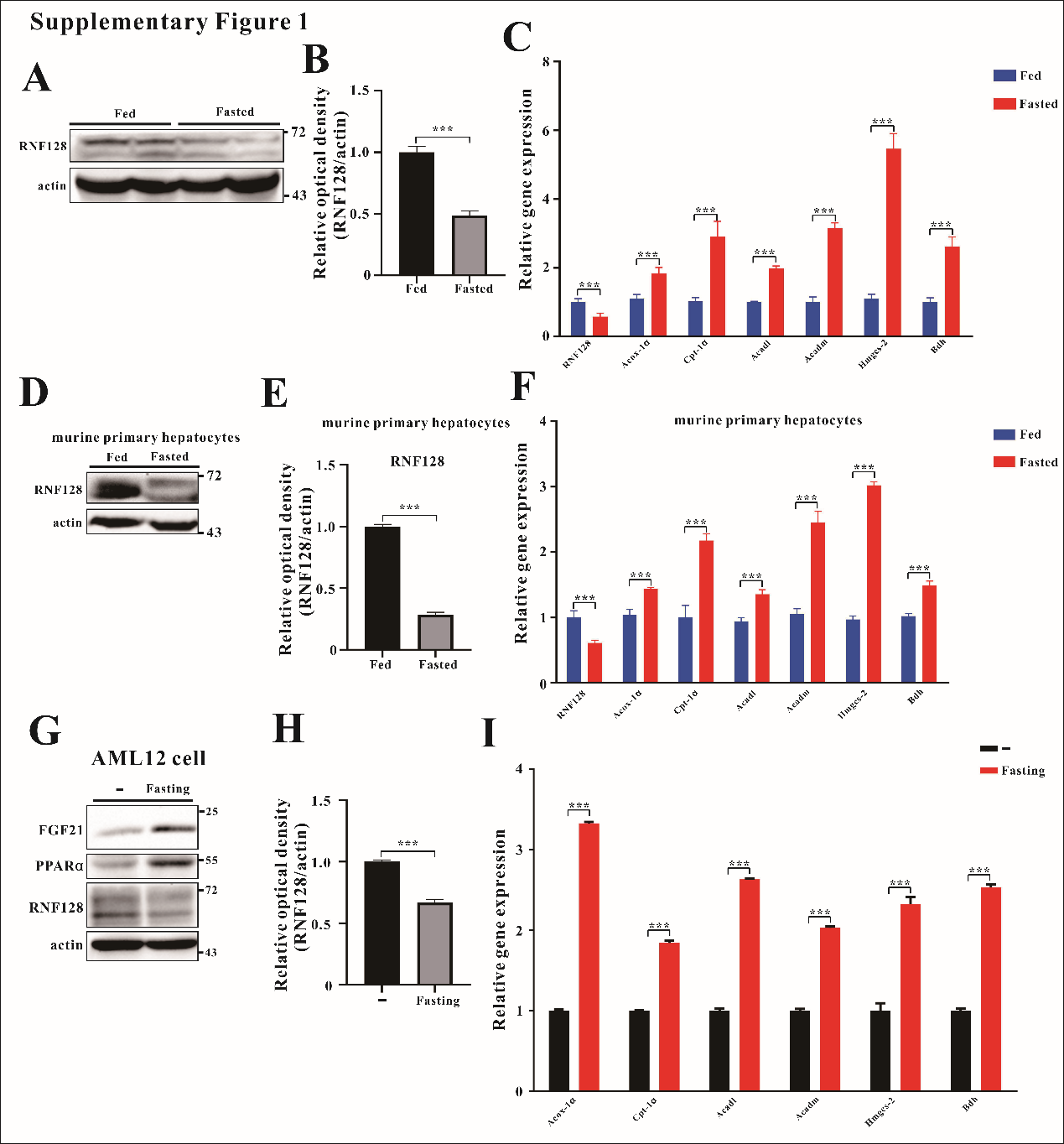
**

**Supplementary Fig. 1** **RING finger protein 128 expression is downregulated in the livers of fasted mice and starved liver cells.**

(A, B) The liver protein levels of RNF128 are measured in mice fed normally or fasted for 24 h. (C) The mRNA expressions of RNF128, peroxisomal acyl-coenzyme A oxidase 1, carnitine palmitoyltransferase 1, acyl-coA dehydrogenase long chain, acyl-coA dehydrogenase medium chain, 3-hydroxy-3-methylglutaryl-coA synthase 2, and 3-hydroxybutyrate dehydrogenase in indicated samples are analyzed using quantitative reverse transcription polymerase chain reaction. (D, E) The protein levels of RNF128 are measured in primary hepatocytes derived from mice fed normally or fasted for 24 hours. (F) The mRNA expressions of RNF128, peroxisomal acyl-coenzyme A oxidase 1, carnitine palmitoyltransferase 1, acyl-coA dehydrogenase long chain, acyl-coA dehydrogenase medium chain, 3-hydroxy-3-methylglutaryl-coA synthase 2, and 3-hydroxybutyrate dehydrogenase in the designated samples are assessed using quantitative reverse transcription polymerase chain reaction. (G, H) The protein levels of RNF128 are measured in AML12 cell lines after treatment with or without fasted medium (contained 1 g/L glucose and 0.5% fetal bovine serum). (I) The mRNA expressions of RNF128, peroxisomal acyl-coenzyme A oxidase 1, carnitine palmitoyltransferase 1, acyl-coA dehydrogenase long chain, acyl-coA dehydrogenase medium chain, 3-hydroxy-3-methylglutaryl-coA synthase 2, and 3-hydroxybutyrate dehydrogenase in the specified samples are quantified using quantitative reverse transcription polymerase chain reaction. Data are presented as the mean ± standard deviation. ^***^*P* < 0.001, Student's *t-*test.

**
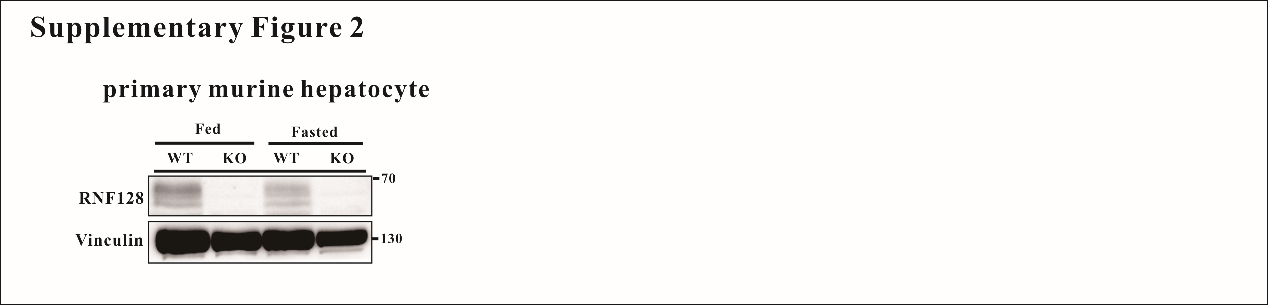
**

**Supplementary Fig. 2 RING finger protein 128 modulates serum insulin and glucagon levels during fasting.** Immunoblots of RNF128 in hepatocytes from WT and RNF128 KO mice after either ad libitum feeding or 24-hour fasting.


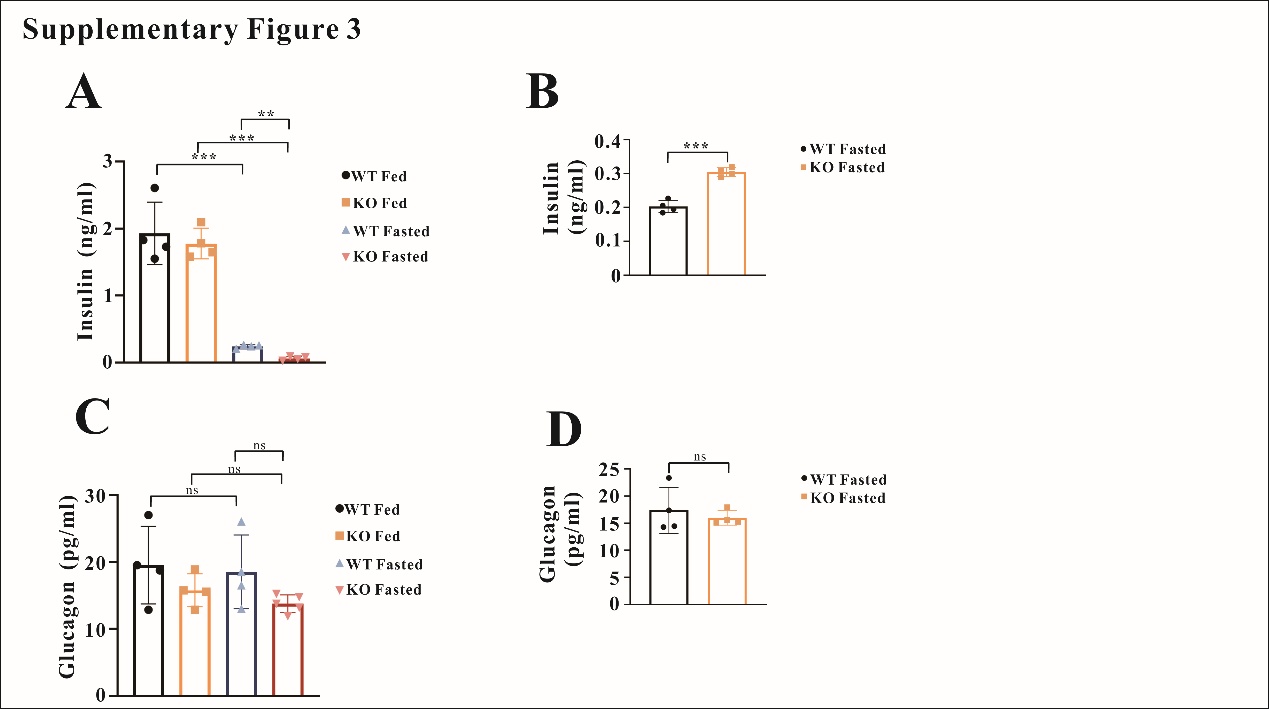


**Supplementary Fig. 3 RING finger protein 128 modulates serum insulin and glucagon levels during fasting.**

(A, C) Analysis of serum insulin, and serum glucagon in the indicated groups following either ad libitum feeding or a 24-hour fasting period (n = 4-5 per group). (B, D) Analysis of serum insulin, and serum glucagon levels obtained from indicated groups after fasting for 24 h (n = 4-5 per group). Data are presented as the mean ± standard deviation. One-way analysis of variance using the Newman–Keuls post hoc test or Student's *t-*test is used to assess the statistical significance. ^**^*P* < 0.01; and ^***^*P* < 0.001.


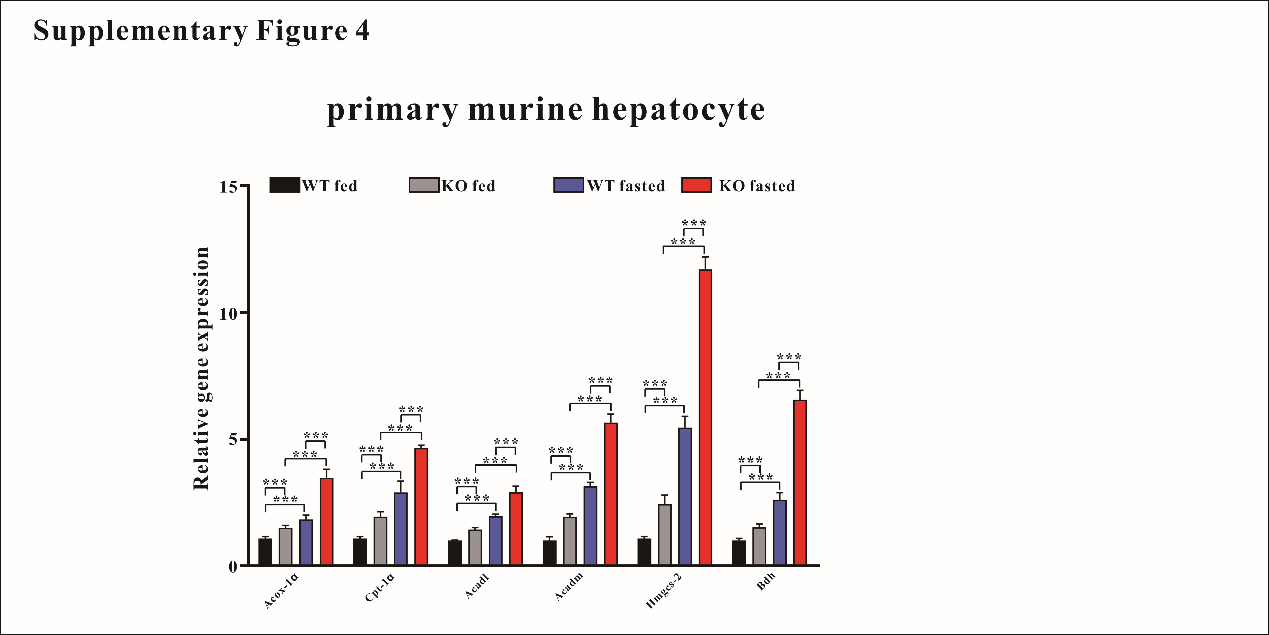


**Supplementary Fig. 4 Expression of genes related to fatty acid β-oxidation and ketogenesis in primary hepatocytes.**

The mRNA expressions of peroxisomal acyl-coenzyme A oxidase 1, carnitine palmitoyltransferase 1, acyl-coA dehydrogenase long chain, acyl-coA dehydrogenase medium chain, 3-hydroxy-3-methylglutaryl-coA synthase 2, and 3-hydroxybutyrate dehydrogenase in indicated samples are analyzed using quantitative reverse transcription polymerase chain reaction. Data are presented as the mean ± standard deviation. One-way analysis of variance using the Newman–Keuls post hoc test or Student's *t-*test is used to assess the statistical significance. ^***^*P* < 0.001.


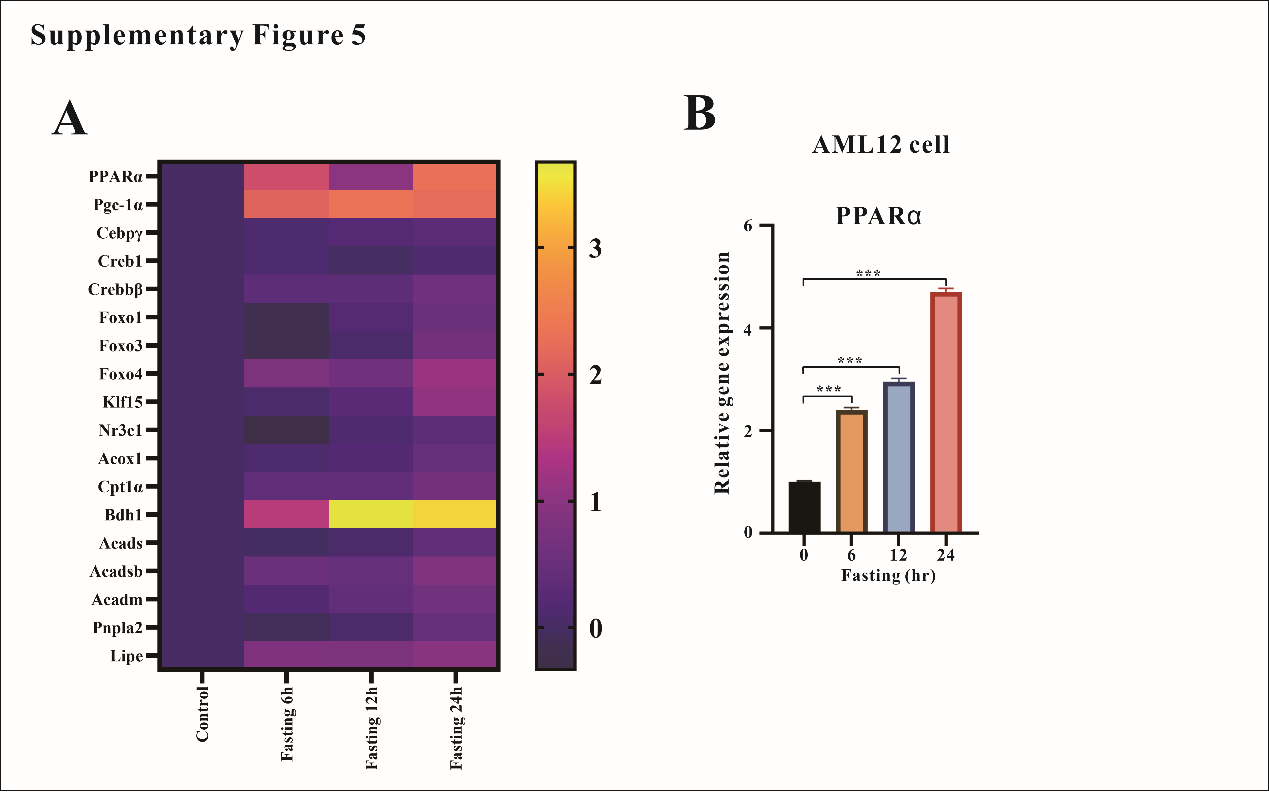


**Supplementary Fig. 5 Peroxisome proliferator-activated receptor alpha** **is upregulated in the starved liver cells.**

(A)The Heat Map of PPARα and fasting-related genes in indicated samples are analyzed using NGS. (B) The mRNA expressions of PPARα in the specified samples are quantified using quantitative reverse transcription polymerase chain reaction. Data are presented as the mean ± standard deviation. ^***^*P* < 0.001, Student's *t-*test.


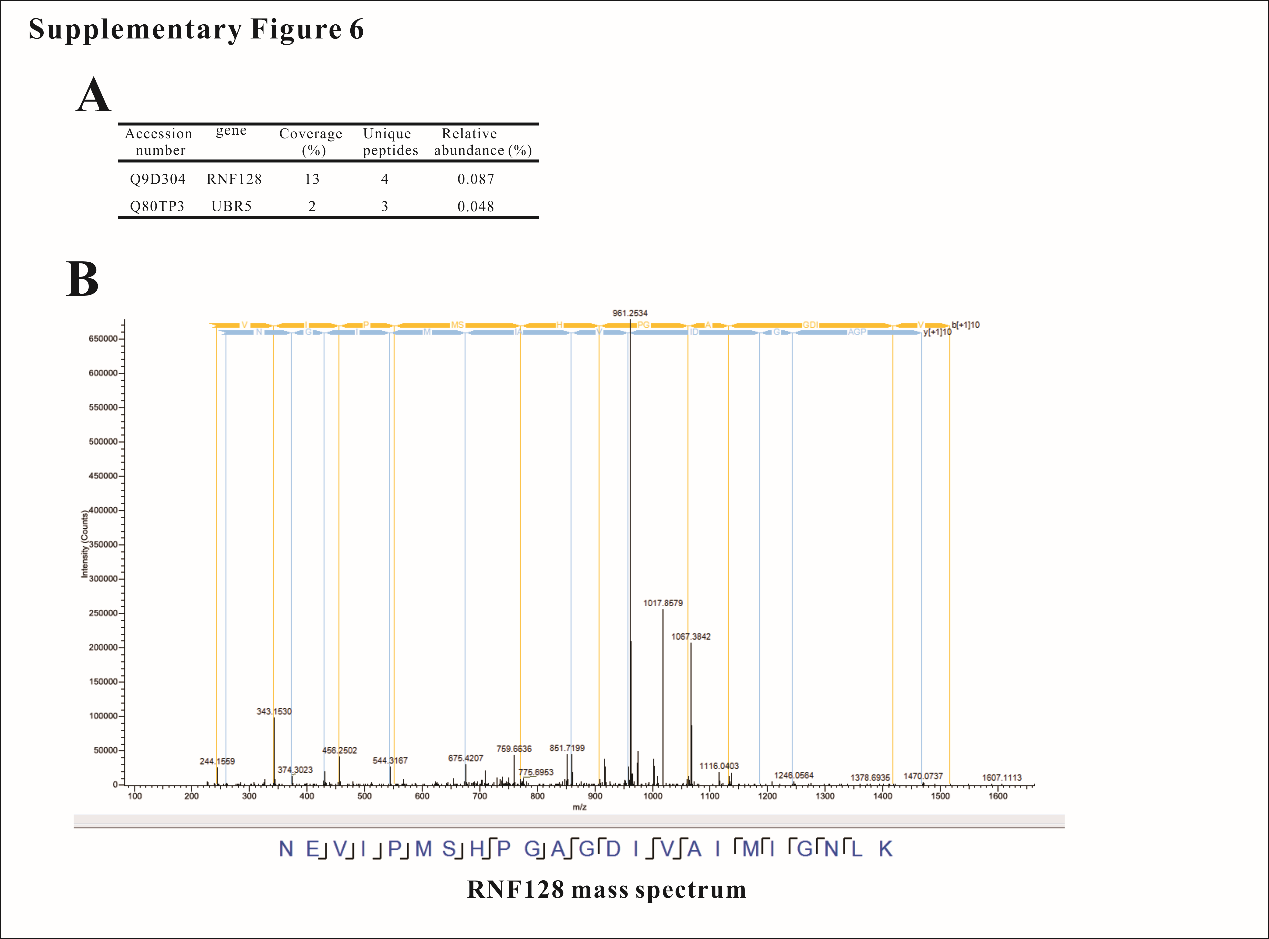


**Supplementary Fig. 6 Co-immunoprecipitation and mass spectrometry analysis of the interaction between RING finger protein 128 and Peroxisome proliferator-activated receptor alpha.**

1. The cell lysates were immunoprecipitated with an anti-PPARα antibody. RNF128 was identified via mass spectrometry. (B**)** Mass spectrometry analysis of RNF128 peptide immunoprecipitated by PPARα.


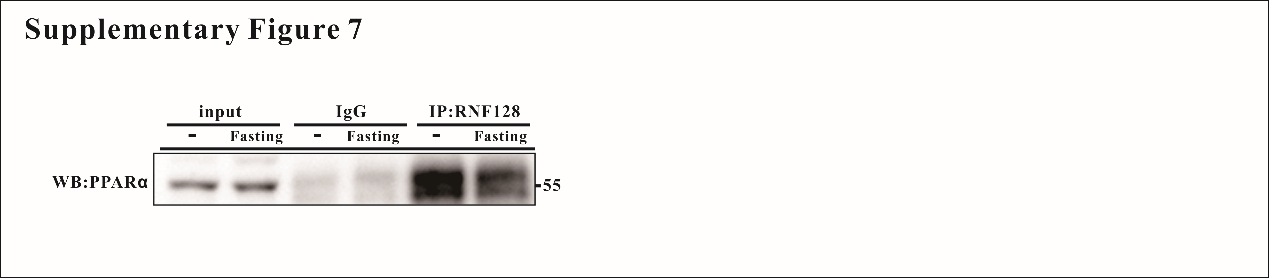


**Supplementary Fig. 7 To evaluate the interaction between RNF128 and** **PPARα during fasting.** Endogenous RNF128 interacts with endogenous PPARα during fasting. Extracts from AML-12 cells are prepared, immunoprecipitated with anti-RNF128, or rabbit anti-immunoglobulin G antibodies, and analyzed using anti-PPARα antibodies.

**
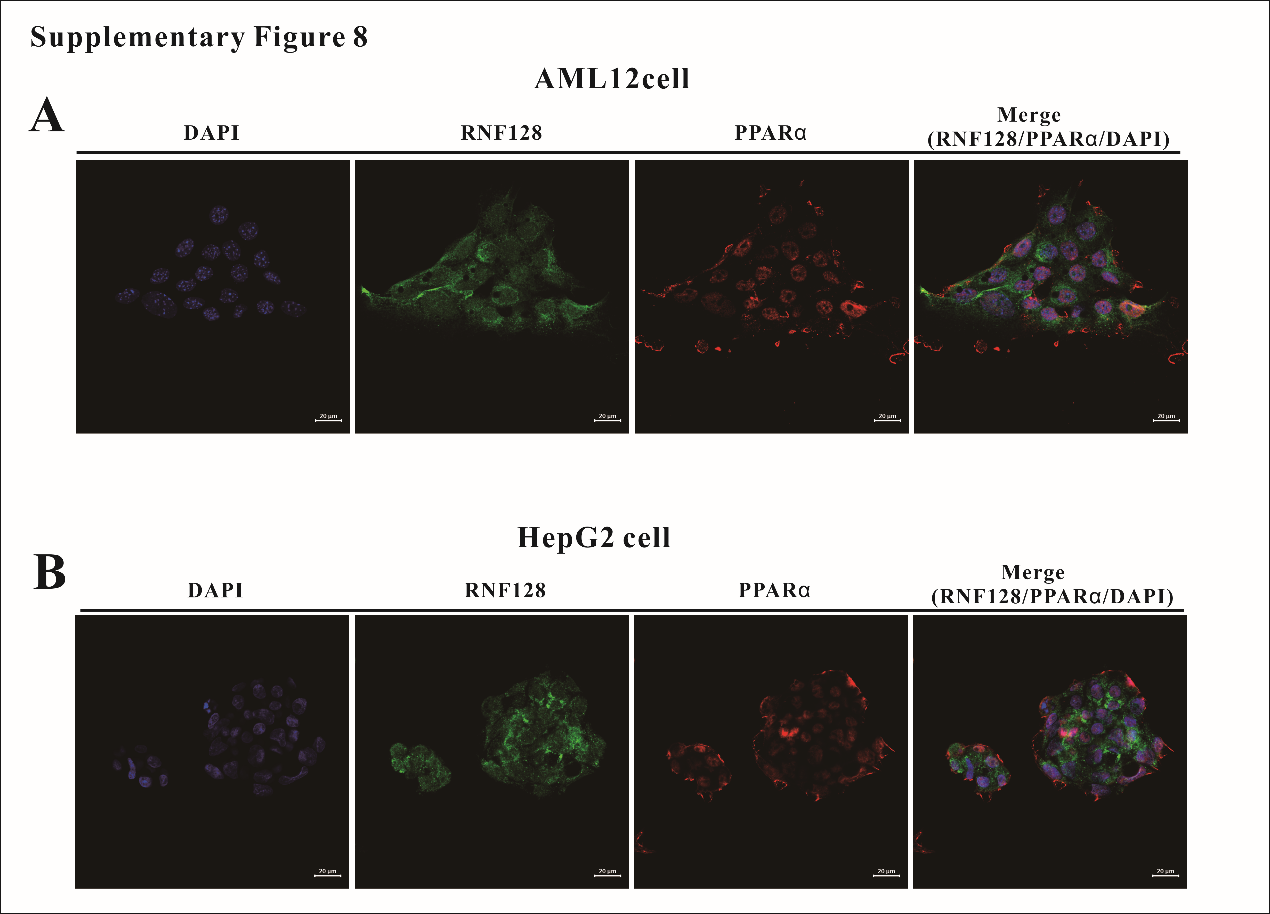
**

**Supplementary Fig. 8 Confocal fluorescence microscopy images of HepG2 and AML12 cells.** (A, B) The subcellular localization of RING finger protein 128 and peroxisome proliferator-activated receptor alpha is analyzed using confocal fluorescence microscopy (LSM880) in the HepG2 and AML12 cells.


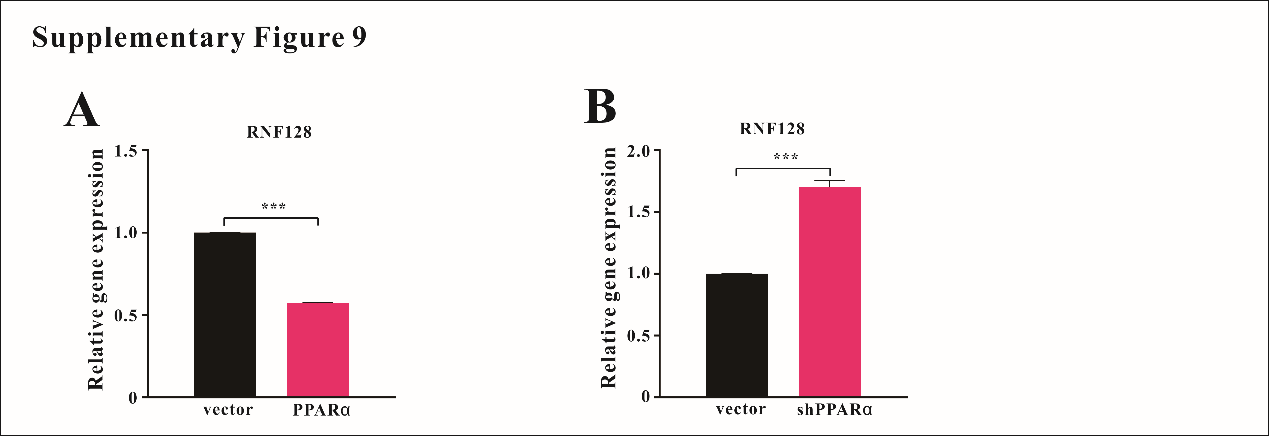


**Supplementary Fig. 9 Peroxisome proliferator-activated receptor alpha regulates the expression of RING finger protein 128 (RNF128) in liver cells.** (A, B) The mRNA expressions of RNF128 in the specified samples are quantified using quantitative reverse transcription polymerase chain reaction. Data are presented as the mean ± standard deviation. ^***^*P* < 0.001, Student's *t-*test.


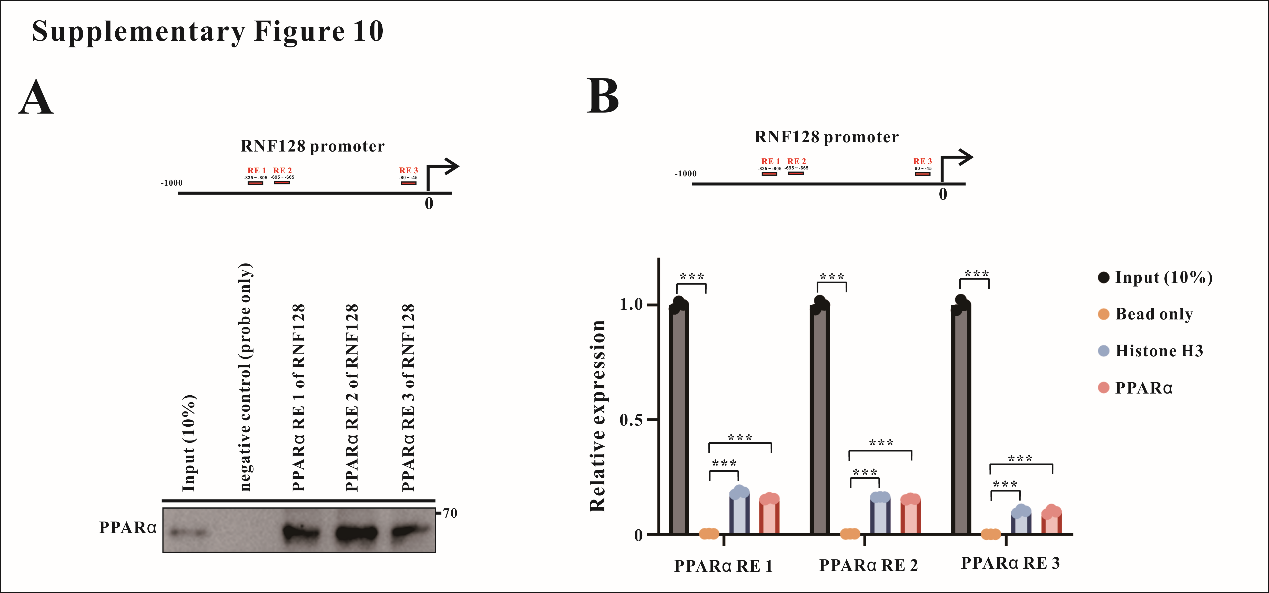


**Supplementary Fig. 10 RNF128 is a PPARα target gene.** (A) Three potential RNF128 PPARα-responsive elements (1, 2 and 3) and a negative control (probe only). Biotin-labeled probes were used to perform the DAPA analysis. (B) AML12 cells were used for ChIP analysis. The eluted DNAs were analyzed by QPCR. Data are presented as the mean ± standard deviation. ^***^*P* < 0.001, Student's *t-*test.


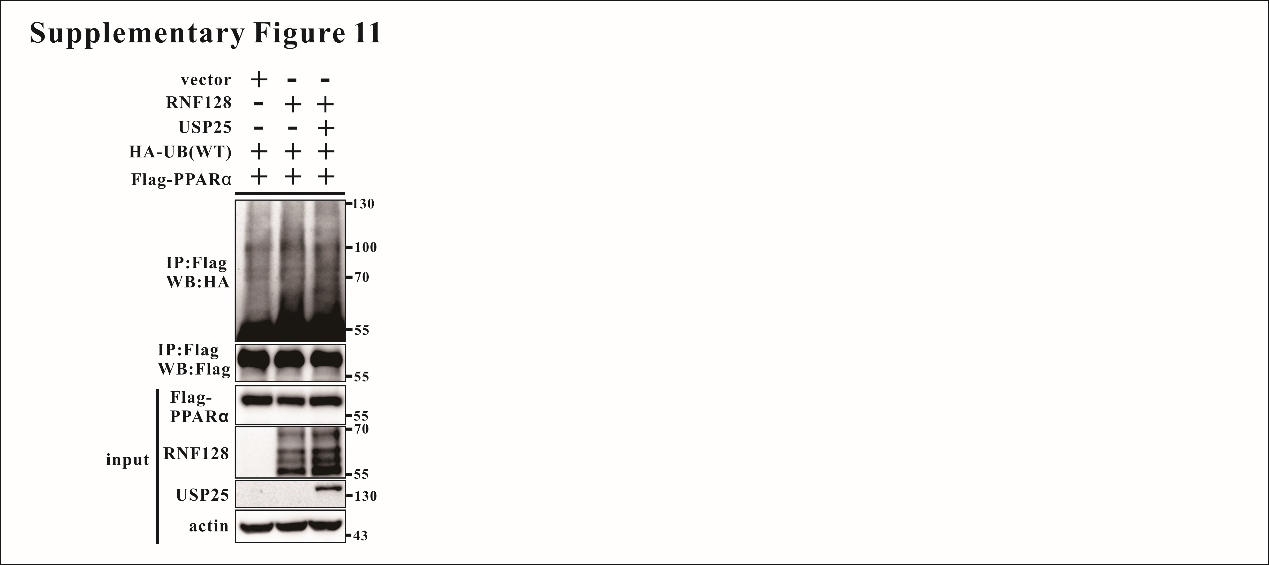


**Supplementary Fig. 11 USP25 decreases the RNF128-mediated polyubiquitination of PPARα.** HEK293 cells are transiently transfected with hemagglutinin-ubiquitin (wild-type), Flag-PPARα, USP25 and RNF128 after 48 h. Lysates are harvested and subjected to immunoprecipitation with Flag antibody and analyzed for ubiquitylation by immunoblotting with the indicated antibody.

| **Supplementary Table 1 \| Primers used for Q-PCR analysis** | | |
| --- | --- | --- |
| **Primers** | **Forward sequence** | **Reverse sequence** |
| **RNF128** | *5’-GCGTCTGGAGCCGTCATCTTTA-3’* | *5’-GGGCCATGTTTTTTCCCTACTTCTAT-3’* |
| **GAPDH** | *5’-TTCACCACCATGGAGAAGGC-3’* | *5’-GATGGCATGGACTGTGGTC-3’* |
| **Human PPARα** | *5’-TCGGCGAGGATAGTTCTGG-3’* | *5’-GATAGCCTGAGGCCTTGTCC-3’* |
| **Human CPT1α** | *5’-ATCAATCGGACTCTGGAAACGG-3’* | *5’-TCAGGGAGTAGCGCATGG -3’* |
| **Human ACOX1α** | *5’-CCGCCGAGAGATCGAGAAC-3’* | *5’-TTCATCAGGGTCAGCGATGC-3’* |
| **Human Acadl** | *5’-CGCCGCGCGATGTTCTC-3’* | *5’-CAGCTTTTTCCCAAACCTCCC-3’* |
| **Human Acadm** | *5’-GGGTTCGGGCGATGCTG-3’* | *5’-ACCAAGTTCCCAGGCTCTTC-3’* |
| **Human Hmgcs-2** | *5’-TGGTCTGTGGAGACATTGCC-3’* | *5’-TATTGGGTACTCCGAGGCCA-3’* |
| **Human Bdh** | *5’-AAGTGAACCTTTGGGGCAC-3’* | *5’-ATCTCATAGCGCAGGCAGTC-3’* |
| **Human FGF21** | *5’-AGATCAGGGAGGATGGGACG-3’* | *5’-AGTGGAGCGATCCATACAGG-3’* |
| **Mouse PPARα** | *5’-GAAAGACCAGCAACAACCCG-3’* | *5’-GGCAAATTCTGTGAGCTCCG-3’* |
| **Mouse CPT1α** | *5’-AAACCCACCAGGCTACAGTG-3’* | *5’-GTAATGTGCGAGCTGCAGTG-3’* |
| **Mouse ACOX1α** | *5’-AGTGCTACGGGTTACATGCC-3’* | *5’-TTACATACGTGCCGTCAGGC-3’* |
| **Mouse Acadl** | *5’-CATCGCAGAGAAACATGGCG-3’* | *5’-TGGCTATGGCACCGATACAC-3’* |
| **Mouse Acadm** | *5’-GAGGAGATTATCCCCGTCGC-3’* | *5’-CACCCCTGTACACCCATACG-3’* |
| **Mouse Hmgcs-2** | *5’-GTGACATTGCAGTCTACCCGA-3’* | *5’-GCACTGGATAGACAGCTTCCC-3’* |
| **Mouse Bdh** | *5’-CATCCTTCAGCCCTGACACC-3’* | *5’-GTCCAGTTCCTTGACCCCAG-3’* |
| **Mouse FGF21** | *5’-GGGGGTCTACCAAGCATACC-3’* | *5’-GGAGACTTTCTGGACTGCGG-3’* |

**Supplementary methods**

**Liquid-chromatography (LC)-MS/MS analysis**

The Samples were digested with modified porcine trypsin at 37°C for 16-hours. The peptides were extracted with 0.1% formic acid and loaded on to a reverse-phase column. The desalted peptides were subjected to LC-MS/MS by using an Orbitrap Elite Hybrid Ion Trap-Orbitrap tandem-mass spectrometer interfaced with an 1D-LC (RP), Dionex Ultimate 3000 RSLCnano system (TOOLS Biotech Company). The data analysis was performed using Proteome Discoverer software (version 1.4, Thermo Fisher Scientific). The MS/MS spectra were searched against the Swissprot database using the Mascot search engine (Matrix Science, London, UK; version 2.5) for peptides identification.
