# Supplementary material for: RNF128 regulates the adaptive metabolic response to fasting by modulating PPARα function
Source: Cell Death Differ. 2025 Sep 10;33(3):512–24. doi: 10.1038/s41418-025-01579-4 (PMC13035816; doi:10.1038/s41418-025-01579-4)

Original western blots for Figure 3

Figure 3B

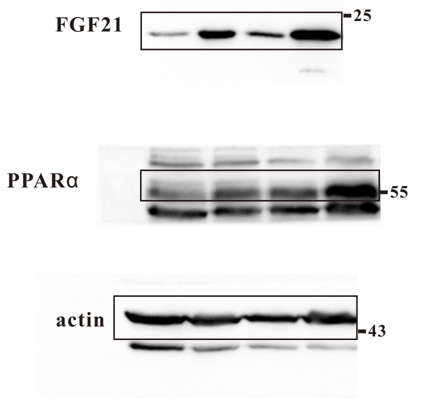

Figure 3F

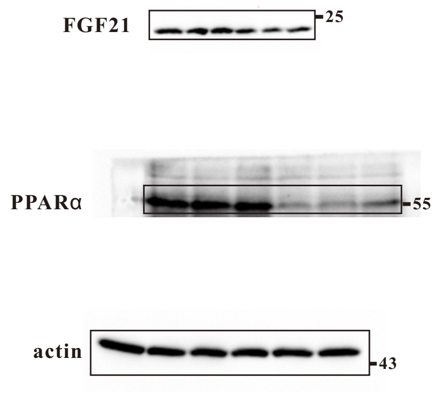

Original western blots for Figure 5

Figure 5A

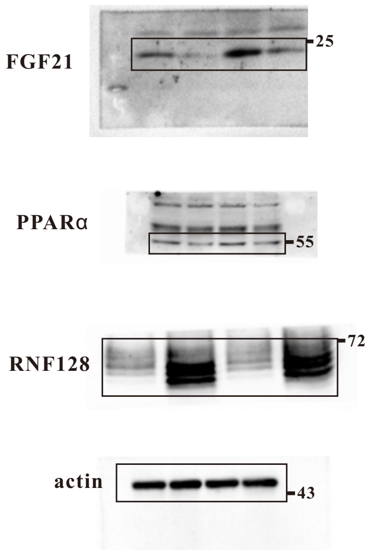

Figure 5C

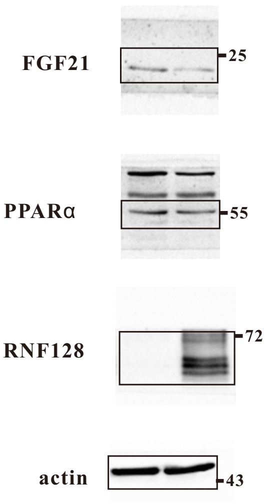

Original western blots for Figure 6

Figure 6A

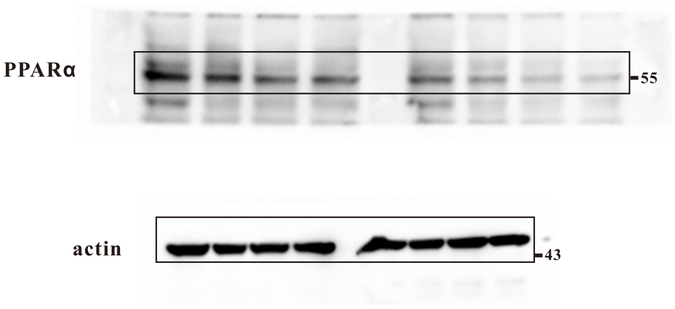

Figure 6C

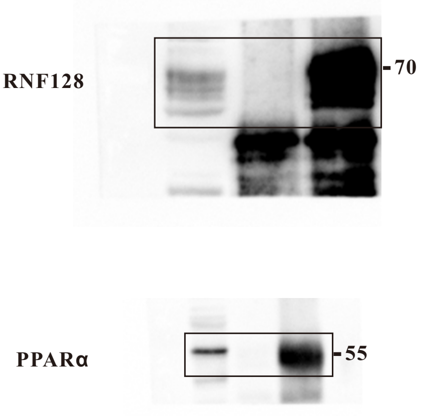

Figure 6D

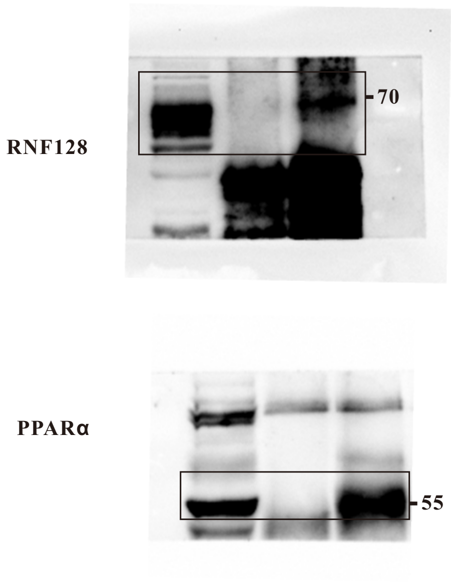

Figure 6E

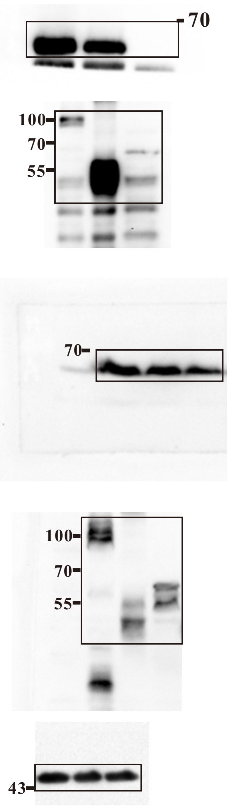

Figure 6F

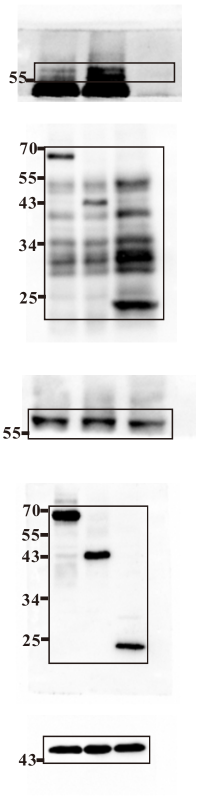

Original western blots for Figure 7

Figure 7A

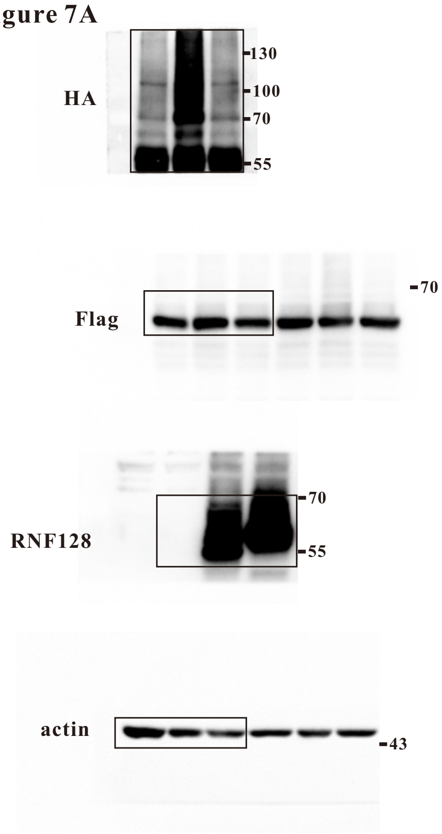

Figure 7B

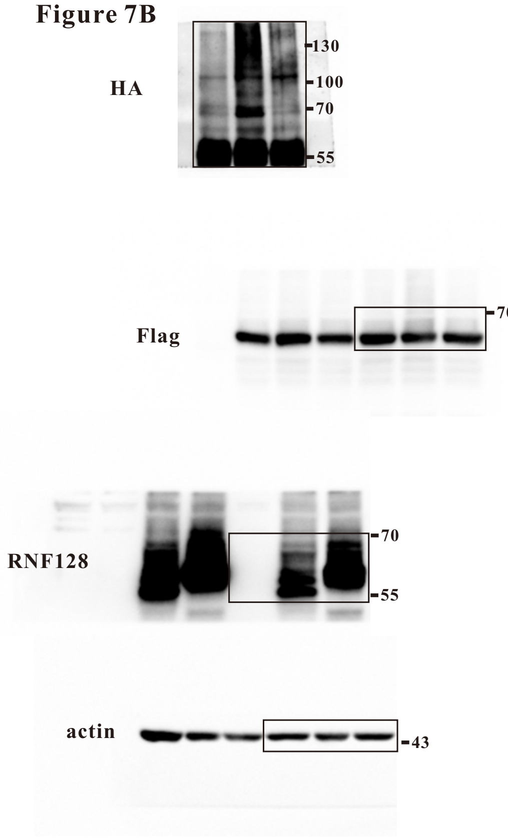

Figure 7C

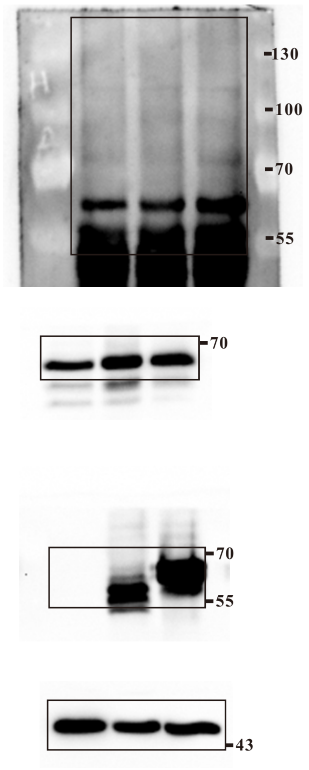

Original western blots for Figure 7

Figure 7D

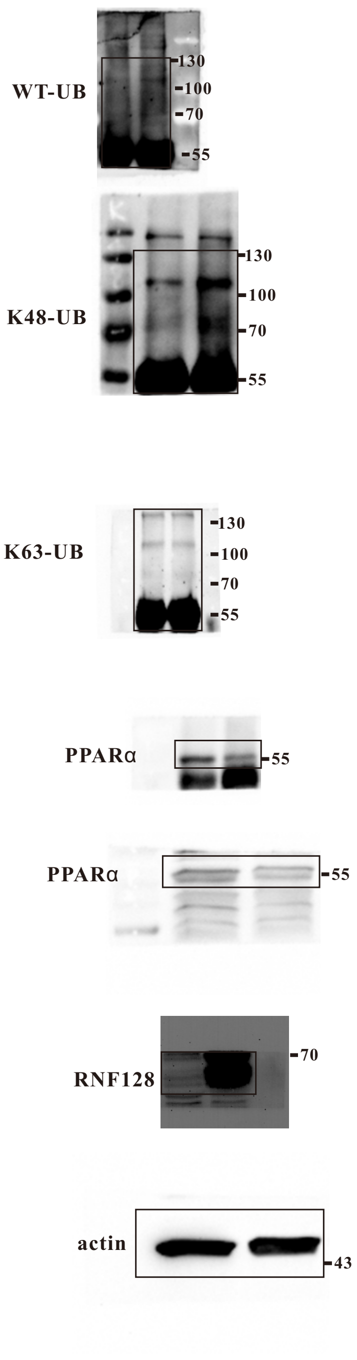

Figure 7E

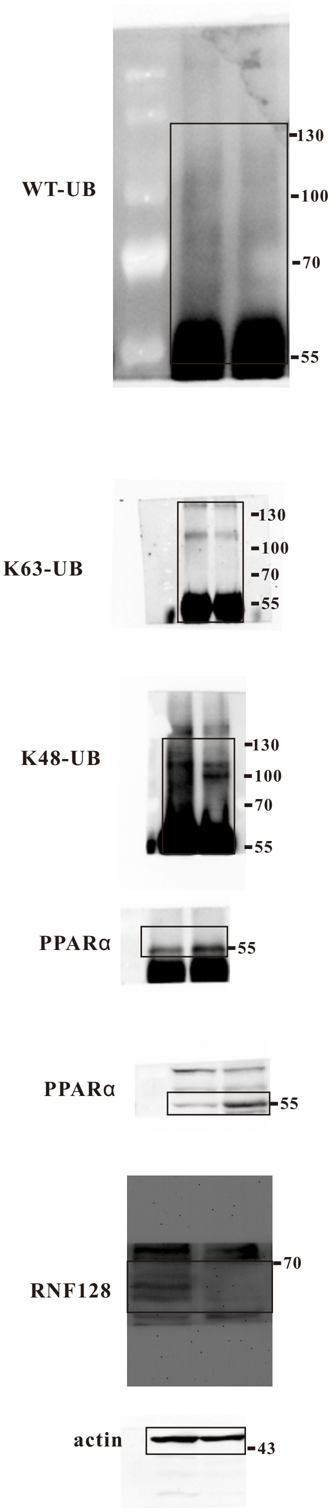

Original western blots for Figure 8

Figure 8A

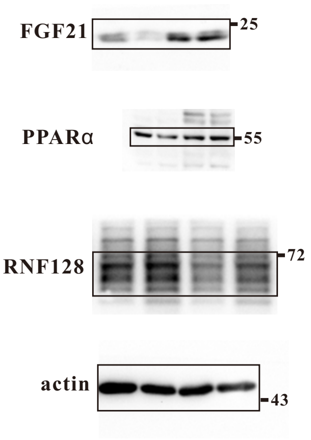

Figure 8B

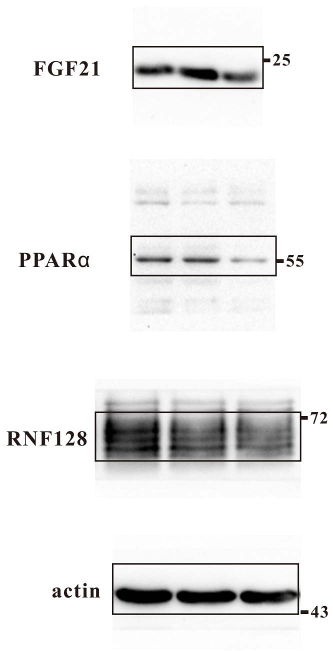

Supplement: Supplementary file 2 — uncropped original western blots [file 41418_2025_1579_MOESM2_ESM.pdf]
